# Cas3-mediated genome reduction: demonstration in *Cupriavidus necator* H16 improves growth on heterotrophic and autotrophic carbon sources

**DOI:** 10.1093/nar/gkag753

**Published:** 2026-08-04

**Authors:** Emily M Fulk, Reuben M Swart, Akira K Nakamura, Laura B Quinto, Violeta Sànchez i Nogué, Christopher H Calvey, Indulekha Tharun, Farren J Isaacs, Christopher W Johnson

**Affiliations:** Renewable Resources and Enabling Sciences Center, National Laboratory of the Rockies, Golden, CO 80401, United States; Renewable Resources and Enabling Sciences Center, National Laboratory of the Rockies, Golden, CO 80401, United States; Department of Molecular, Cellular, and Developmental Biology, Yale University, New Haven, CT 06511, United States; Systems Biology Institute, Yale University, West Haven, CT 06516, United States; Department of Molecular, Cellular, and Developmental Biology, Yale University, New Haven, CT 06511, United States; Systems Biology Institute, Yale University, West Haven, CT 06516, United States; Renewable Resources and Enabling Sciences Center, National Laboratory of the Rockies, Golden, CO 80401, United States; Renewable Resources and Enabling Sciences Center, National Laboratory of the Rockies, Golden, CO 80401, United States; Renewable Resources and Enabling Sciences Center, National Laboratory of the Rockies, Golden, CO 80401, United States; Department of Molecular, Cellular, and Developmental Biology, Yale University, New Haven, CT 06511, United States; Systems Biology Institute, Yale University, West Haven, CT 06516, United States; Department of Biomedical Engineering, Yale University, New Haven, CT 06511, United States; Renewable Resources and Enabling Sciences Center, National Laboratory of the Rockies, Golden, CO 80401, United States

## Abstract

Genome reduction is widely used to improve microbial bioprocessing hosts by reducing the burden of inessential physiology. Rationally identifying genomic regions that are dispensable or even detrimental to bioprocessing is challenged by our inability to map genome sequence to function across complex regulation and physiology. Thus, there is a need for tools that rapidly generate reduced genome strains with improved performance in process-relevant conditions. Here, we report a Cascade–Cas3-enabled method called TRIM3 that generates large deletions by targeting a randomly integrated transposon, enabling facile generation of a genome-reduced mutant library. Mutants with improved performance were isolated following growth-coupled selection and analyzed by long-read DNA sequencing to identify deletions in their genomes. We deploy this system iteratively in the industrial host *Cupriavidus necator* H16 on fructose and on formate. After two rounds of TRIM3, we isolate a strain containing a total reduction of 1.4 Mb (18.4% of the genome) that grows 25% faster in a bioreactor on fructose and a strain with a total reduction of 0.5 Mb (7.3% of the genome) that grows 14% faster on formate. This work demonstrates a method for random, iterative, growth-selectable genome reduction that represents a new avenue for large-scale genome modifications and the development of improved bioprocessing hosts.

## Graphical Abstract

**Graphical Abstract:**
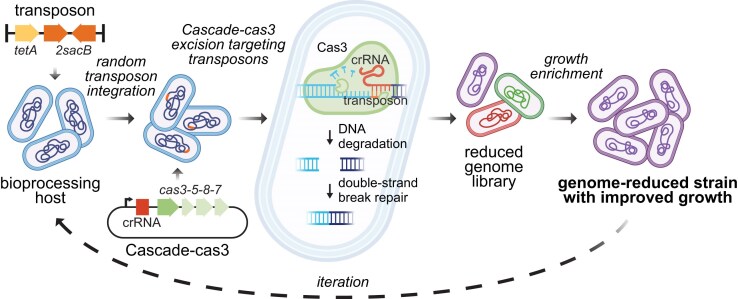

## Introduction

As the bioeconomy grows, there is an increasing interest in developing microbial strains optimized for robust growth and performance under specific bioprocessing conditions rather than in their natural environment. Top-down genome reduction, undertaken by identifying and removing presumably nonessential genomic regions, is an attractive strategy for improving industrial host performance [1, 2] and has in some cases resulted in strains with improved growth rates [3–6], increased yield of biomass [3, 4, 6–8] and production of heterologous products [3, 4, 6, 7, 9–12], improved genetic tractability [5, 6, 9, 11, 13], and improved stress tolerance [12]. However, rational reductions have not always proved beneficial; deletion of putatively unnecessary genes has sometimes resulted in strains that either perform comparatively to or worse than the parent in terms of growth [7, 10, 13–16] or product production [13, 16], or resulted in other undesirable phenotypes such as the loss of fitness in nutrient-limited or stressful conditions [4, 14]. To overcome challenges in rationally identifying beneficial deletions, coupling random deletions to growth-based selection shows promise for rapid identification of genome reductions that confer fitness benefits under the desired conditions [17]. In the past several years, systems that combine transposon mutagenesis with DNA nucleases or recombinases have successfully generated pools of cells with random genome reductions [17–19]. In these strategies, transposons carrying DNA targets such as *loxP* sites [18], a CRISPR-Cas9 targeting sequence [19], or an I-SceI meganuclease cleavage site [17] are randomly inserted into the host strain genome. Activities of the transposon-targeting machinery—Cre recombinase [18], Cas9 [19], or I-SceI [17]—result in recombination that removes fragments of the genome to enable cell survival. In some cases, a counterselectable marker on the transposon helps to select against cells that have not lost the transposon through genomic deletions [17]. These strategies highlight the potential for selection-coupled genome reduction to generate streamlined microbial hosts with performance advantages under bioprocessing conditions.

*Cupriavidus necator* (previously named *Ralstonia eutropha, Alcaligenes eutrophus, Wautersia eutropha*, and *Hydrogenomonas eutropha*) is a gram-negative bacterium that shows great promise as a biomanufacturing host for conversion of diverse waste streams into value-added products. Originally isolated from soil, *C. necator* H16 (hereafter *C. necator*) is notable for its metabolic flexibility and can utilize a wide variety of carbon sources and energy sources [20–22]. It can grow autotrophically by fixing CO_2_ through the Calvin–Benson–Bassham (CBB) cycle and oxidizing H_2_ for energy [23], or heterotrophically by metabolizing substrates including organic acids [24–26], aromatic compounds [27, 28], and fructose [25]. Its ability to grow lithoautotrophically on CO_2_, or organoautotrophically on formate, which can be produced by electrochemically reducing CO_2_, make it particularly attractive as a platform organism for waste carbon valorization [29]. As a host for value-added product production, *C. necator* has been grown industrially as a natural producer of polyhydroxyalkanoates for biopolymers [30–32] and has more recently been engineered to produce a wide variety of heterologous compounds, including acetoin [33], terpenoids [34, 35], polyphenols [36], alkanes and alkenes [37], alcohols [38], fatty acids [39–41], amino acids [42, 43], sugars [44–46], and sugar alcohols [47, 48]. Its metabolic versatility, along with availability of genome-editing tools and characterized genetic parts [49–51], make it an attractive host for strain engineering efforts.

While its generalist lifestyle serves *C. necator* in dynamic natural environments and makes it a promising platform host for valorizing a wide variety of substrates, it is not optimized for growth or product production under defined bioprocessing conditions. The genome of *C. necator* is large (7.4 Mb) and consists of three replicons: chromosome 1 (4.1 Mb), which encodes essential housekeeping functions and genes for growth on heterotrophic substrates, chromosome 2 (2.9 Mb), which carries additional genes for secondary metabolism and substrate utilization, including one copy of the CBB operon, and the pHG1 megaplasmid (0.5 Mb), which carries genes for lithoautotrophic growth including hydrogenase genes and a second copy of the CBB operon [20, 52]. Proteomic studies have estimated that over 40% of the proteome is underused in a bioreactor environment or is of unknown function, suggesting that the cell expends unnecessary resources on proteins not needed for growth [53]. Transposon mutagenesis studies have shown benefit for reducing protein synthesis burden through gene disruption [54]. Adaptive laboratory evolution (ALE) on formate resulted in 12–142 kb deletions on the pHG1 megaplasmid and removal of pHG1 entirely (6% of the genome) improved growth on multiple substrates [55]. A second ALE study also found that loss of pHG1 improved growth on formate [56]. Taken together, these findings demonstrate that genome reduction is a promising strategy for improving the growth of *C. necator* through genomic streamlining.

In this work, we develop Transposon-mediated Random Iterative Minimization with Cascade–Cas3 (TRIM3), a novel strategy for generating improved strains through random genome reduction coupled with growth selection, and apply this strategy to generate reduced-genome strains of *C. necator* with improved growth on fructose and formic acid. We first design and evaluate a cassette containing strong selectable and counterselectable markers and use this selection scheme to generate a *C. necator* transposon library. Next, we induce expression of a Type I-C Cascade–Cas3 system. Unlike Class 2 CRISPR systems such as Cas9 and Cas12a, which make double-strand breaks that can result in small deletions upon repair, Cas3 is a helicase-nuclease enzyme that cuts and degrades DNA bidirectionally, which can result in large deletions upon repair [57]. By targeting Cas3 to the transposon and counterselecting for its excision, we can generate a mutant pool containing large genome deletions. We then enrich for cells with improved growth on fructose or formic acid in a bioreactor and identify *C. necator* isolates with 2.0%–14.9% genome reductions after one round of selection. Subjecting these isolates to a second round of library generation and selection identified *C. necator* isolates with 1.4 Mb (18.4%) total genome reduction relative to the wild-type parent on fructose and 0.5 Mb (7.3%) genome reduction on formate. Compared to the parent strain, we show that these isolates grow 25% and 14% faster on fructose and formate in bioreactors, respectively. Overall, this work leverages the recent technological advances of Cascade–Cas3 and long-read sequencing in a new strategy for random, iterative genome reduction to improve growth of biomanufacturing hosts in process-relevant conditions.

## Materials and methods

### Plasmid construction

All plasmids constructed in this study, along with their complete annotated sequences, are available from Addgene (www.addgene.org). Annotated plasmid sequences in GenBank format are also included in the online supplementary material. A list of plasmids used in this study, along with their Addgene plasmid #, can be found in Table 1. To build plasmids for inserting chloramphenicol and tetracycline resistance cassettes into the *C. necator* genome, the chloramphenicol acetyltransferase (*cat*) gene was amplified by polymerase chain reaction (PCR) from pBTL-3 [58] (Addgene plasmid #22807) and the tetracycline efflux pump (*tetA*) was amplified from pCasPA [59] (Addgene plasmid #113347). Each fragment was assembled into a pK18msB [60] (Addgene plasmid #177839) backbone containing ≈ 750 bp homology arms flanking the *phaCAB* locus in the *C. necator* H16 genome, yielding plasmids pEMF009 (*cat*, Addgene plasmid #254852) and pEMF012 (*tetA*, Addgene plasmid #254853). Plasmids for generating transposon libraries, pEMF044 (Addgene plasmid #242105), pEMF053 (Addgene plasmid #242106), and pEMF054 (Addgene plasmid #242107), were built using the pKMW7 backbone [61], a gift from Adam Deutschbauer (Joint Genome Institute), assembled with PCR-amplified fragments for tetracycline resistance and *sacB* expression via either Gibson assembly or Golden Gate assembly. *tetA* was amplified from pBMTL-4 [58] (Addgene plasmid #22814), and *sacB*-*1* was amplified from pK18msB [60] (Addgene plasmid #177839). *sacB-2* was codon-optimized for *C. necator* by BaseBuddy [62] using the *match_codon_usage* method and synthesized by Twist Bioscience. Plasmids pEMF047 (Addgene plasmid #242173) and pEMF049 (Addgene plasmid #242190) for expressing Cascade–Cas3 in *C. necator* were assembled from PCR-amplified *rhaSR* and P*_rhaB:_*:crRNA:*cas3587* from pCas3cRh [57] (Addgene plasmid #133773) with the PCR-amplified pBBR1 *ori* from pBMTL-4 [58] (Addgene Plasmid #22814) and the kanamycin resistance marker from pK18msB (Addgene plasmid #177839) [60] via HiFi DNA assembly (NEB, E2621). To add the CRISPR RNA (crRNA) sequence targeting *sacB*-*1*, the *C. necator* Cascade–Cas3 plasmid was digested with BsaI and ligated with duplexed oligos coding for the target sequence by T4 DNA ligase. Oligos were synthesized by Integrated DNA Technologies, and fragments were amplified using Platinum SuperFi II DNA polymerase (Invitrogen, 12361010). All assemblies were transformed into *Escherichia coli* 5-alpha F’*I*^*q*^ (New England Biolabs, C2992) or *E. coli* One Shot PIR2 (for plasmids with the R6K γ ori) (Invitrogen, C111110) and were selected on Luria broth (LB) (Miller) agar plates supplemented with 50 µg/ml kanamycin. Correct plasmid sequences were verified by nanopore sequencing (Plasmidsaurus).

**Table 1.** Plasmids used in this study

Plasmid	Description	Source	Addgene plasmid #
pBTL-3	Broad host range vector; source of *cat* for pEMF009	Lynch *et al*., 2006 [58]	22807
pCasPA	Cas9 expression vector; source of *tetA* for pEMF012	Chen *et al*., 2018 [59]	113347
pK18msB	Plasmid for gene replacement using kanamycin/sucrose selection/counterselection; source of *sacB-1* for pEMF044 and pEMF054	Ling *et al*., 2022 [60]	177839
pEMF009	Plasmid for integration of *cat* into the Δ*phaCAB* locus in *C. necator* H16	This study	254852
pEMF012	Plasmid for integration of *tetA* into the Δ*phaCAB* locus in *C. necator* H16	This study	254853
pBMTL-4	Broad host range vector; source of *tetA* for pEMF044, pEMF053, and pEMF054	Lynch *et al*., 2006 [58]	22814
pKMW7	Nonreplicative plasmid for random insertion of a Tn5 transposon	Wetmore *et al*., 2015 [61]	N/A
pEMF044	Nonreplicative plasmid for random insertion of a Tn5 transposon with *tetA* and *sacB-1*	This study	242105
pEMF053	Nonreplicative plasmid for random insertion of a Tn5 transposon with *tetA* and codon-optimized *sacB-2*	This study	242106
pEMF054	Nonreplicative plasmid for random insertion of a Tn5 transposon with *tetA and* 2*sacB*	This study	242107
pCas3cRh	Replicative plasmid for rhamnose-inducible expression of Cascade–Cas3 and crRNA; source of *rhaSR* and P*_rhaB:_*:crRNA:*cas3587* for pEMF047 and pEMF049	Csörgő *et al*., 2020 [57]	133773
pEMF047	Replicative plasmid for rhamnose-inducible expression of Cascade–Cas3	This study	242173
pEMF049	Replicative plasmid for rhamnose-inducible expression of Cascade–Cas3 with crRNA targeting *sacB-1*	This study	242190

### Strain cultivation

For general *C. necator* growth in liquid cultures, strains were cultivated in a mix of 50% v/v minimal salts medium (MSM) (3.746 g/l K_2_HPO_4_, 1.156 g/l KH_2_PO_4_, 0.962 g/l NH_4_Cl, 0.702 g/l NaCl, 66 mg/l citric acid, 16.68 mg/l FeSO_4_⋅7H_2_O, 0.1 mg/l ZnCl_2_, 0.03 mg/l MnCl_2_⋅4H_2_O, 0.05 mg/l CoCl_2_⋅6H_2_O, 0.07 mg/l CuCl_2_⋅2H_2_O, 0.12 mg/l NiCl_2_⋅6H^2^O, 0.03 mg/l Na_2_MoO_4_⋅2H_2_O, 0.05 mg/l CrCl_3_⋅6H_2_O, 0.3 mg/l H_3_BO_3_, 11 mg/l CaCl_2_, and 240 mg/l MgSO_4_) [55] supplemented with 10 g/l fructose and 50% v/v LB (Miller) (LB + FN10). For growth on plates, *C. necator* was grown on either LB (Miller) with 15 g/l agar or on YTS plates (5 g/l yeast extract, 10 g/l tryptone, 150 g/l sucrose, and 15 g/l agar) for sucrose counterselection. Where required, *C. necator* cultures were supplemented with 200 µg/ml kanamycin (Kan200), 15 µg/ml gentamicin (Gent15), or 10 μg/ml tetracycline (Tet10). Unless otherwise noted, cultures were incubated at 30°C with 225 revolutions per minute (rpm) shaking for liquid cultures. *Escherichia coli* cultures were cultivated in LB (Miller) (+15 g/l agar for plates) with 50 μg/ml kanamycin (Kan50) at 37°C, with 225 rpm shaking for liquid cultures. A list of all strains in this study can be found in Table 2.

**Table 2.** Strains used in this study

Strain	Description	Source
*Escherichia coli* S17-1	Conjugation donor strain for Cascade–Cas3 plasmid	Simon *et al*., 1983 [63]
*Escherichia coli* WM6026	Conjugation donor strain containing the *pir* gene for Tn5 transposase plasmids; 2,6-diaminopimelic acid auxotroph	Blodgett *et al*., 2007 [64]
*Cupriavidus necator* CHC023	*Cupriavidus necator* H16 (ATCC 17699) ΔH16_A0006 Δ*phaCAB*	Calvey *et al*., 2023 [55]
*Cupriavidus necator* CHC123	*Cupriavidus necator* H16 (ATCC 17699) ΔH16_A0006 Δ*phaCAB* ΔpHG1	Calvey *et al*., 2023 [55]
*Cupriavidus necator* EMF069	*Cupriavidus necator* H16 (ATCC 17699) ΔH16_A0006 Δ*phaCAB::cat*	This study
*Cupriavidus necator* EMF070	*Cupriavidus necator* H16 (ATCC 17699) ΔH16_A0006 Δ*phaCAB::tetA*	This study

TRIM3 isolates are described in Supplementary Tables S1 and S2.

### Evaluating counterselectable markers

To evaluate the survival rate of cells carrying *sacB* markers when selected on sucrose, *E. coli* donor cells carrying the transposon plasmid were conjugated with CHC023 as described previously [60]. *Cupriavidus necator* transconjugants with the transposon successfully integrated into the genome were selected by at least two successive selections on LB-Tet10-Gent15 plates. Each transposon library was scraped and grown overnight in LB-FN10-Gent15-Tet10 liquid culture, adjusted to OD_600 _= 5, and plated in serial dilutions on nonselective LB plates and counterselective YTS plates. After 48 h of growth at 30°C, colonies from each condition were counted and the escape frequency was calculated as the ratio of colonies observed on YTS plates compared to colonies observed on LB. To elucidate the mechanism of sucrose escape, the transposon was PCR amplified from escape isolates and the purified PCR products were sequenced by nanopore amplicon sequencing (Plasmidsaurus). Isolates that did not result in an amplified transposon product were sequenced using nanopore whole-genome sequencing.

### Construction of genome-reduced libraries with TRIM3

To generate *C. necator* transposon libraries, *E. coli* WM6026 donor cells carrying pEMF054 (*tetA* + 2*sacB*) were conjugated with CHC023 or CHC123 on LB plates supplemented with 300 µM 2,6-diaminopimelic acid for 24–48 h. Transconjugants were selected by scraping populations from the conjugation plate onto a first LB-Tet10-Gent15 plate and incubating for 48–72 h. The entire transposon library was scraped from the first selection plate and plated onto a second LB-Tet10-Gent15 plate to encourage propagation of the transposon through every copy of the genome. After growth of the second selection plate for 48–72 h, transposon libraries were scraped and pooled. Aliquots of the pooled transposon library were either glycerol stocked for future use or used to directly inoculate 50 ml LB-FN10-Tet10-Gent15 in a 250 ml baffled shake flask and grown overnight. The transposon libraries were conjugated with *E. coli* S17-1 donor cells carrying pEMF049 (*sacB-1* crRNA + Cascade–Cas3), plated on LB, and incubated for 24–48 h. Transconjugants were selected once on LB-Kan200-Gent15 plates incubated for 24–48 h. These plates were scraped and inoculated to OD_600 _≈ 0.3 in LB-Kan200-Gent15 medium supplemented with 10 mM L-rhamnose to induce Cascade–Cas3 expression. After 24 h of growth, cultures were adjusted to OD_600 _= 5–10, plated on first YTS-Gent15 plates, and incubated for 48–72 h to select for sucrose-resistant colonies. These populations were scraped, plated onto a second YTS-Gent15 plate, and incubated for 24–48 h. The second YTS-Gent15 plates were scraped, and this population used to inoculate seed cultures for bioreactor selections or made into glycerol stocks containing 20% glycerol for later inoculations.

### Mapping of transposon insertion sites

To map the transposon insertions in the libraries prior to introduction of Cascade–Cas3, a 1 ml glycerol stock of each transposon library (normalized to an OD_600_ of 50) was first thawed at room temperature. To lyse the cells, the libraries were resuspended in 10 ml of extraction buffer (100 mM Tris, pH 8, 100 mM ethylenediaminetetraacetic acid, pH 8, 1.4 M NaCl) and incubated with 50 mg lysozyme and 1 µg RNase A at 37°C for 20 min, followed by incubation with 1.2 ml of 20% sodium dodecyl sulfate and 1 µg proteinase K at 56°C for 2 h. Lysate was centrifuged at 2300 x *g* for 5 min and supernatant was kept and adjusted to 20 ml with 10 mM Tris, pH 8.0. For DNA extraction, 15 ml chloroform was added to the lysate supernatant, centrifuged at aforementioned parameters, and the aqueous layer was washed with 15 ml chloroform twice. 1.5 ml 3 M sodium acetate, pH 5.2, was added, followed by 15 ml isopropyl alcohol. The samples were placed in −20°C for 15 min to promote DNA precipitation and then spun at 20,000 x *g* for 30 min to pellet DNA. The DNA pellet was washed with 70% ethanol and resuspended in 250 µl Tris, pH 8, overnight. DNA sample concentrations were normalized and custom Tn-seq library preparation (protocol adapted from Jahn *et al*., 2021 [53] but using a biotinylated primer with the sequence /5BiosG/CGTTCTTCTGCTGCTCGGGGATCTGG) and 150 bp paired-end short-read sequencing on an Element AVITI benchtop sequencing instrument were performed by AmpSeq. Sequencing data were analyzed to map transposon insertion sites using scripts and parameters available at https://github.com/akirakaren/map_Tninsertions.

### Growth enrichments

To enrich the genome-reduced libraries for cells with improved growth, cells were inoculated into baffled shake flasks containing LB-FN10-Gent15 at an OD_600 _≈ 0.3 and grown overnight for ≈ 16 h. Overnight cultures were spun down at 4000 rpm for 10 min and cell pellets resuspended in MSM containing 3× concentration of trace micronutrients (3.746 g/l K_2_HPO_4_, 1.156 g/l KH_2_PO_4_, 0.962 g/l NH_4_Cl, 0.702 g/l NaCl, 198 mg/l citric acid, 50.04 mg/l FeSO_4_⋅7H_2_O, 0.3 mg/l ZnCl_2_, 0.09 mg/l MnCl_2_⋅4H_2_O, 0.15 mg/l CoCl_2_⋅6H_2_O, 0.21 mg/l CuCl_2_⋅2H_2_O, 0.36 mg/l NiCl_2_⋅6H_2_O, 0.09 mg/l Na_2_MoO_4_⋅2H_2_O, 0.15 mg/l CrCl_3_⋅6H_2_O, 0.9 mg/l H_3_BO_3_, 11 mg/l CaCl_2_, and 240 mg/l MgSO_4_) and supplemented with either 20 g/l fructose or 1.36 g/l (20 mM) sodium formate. These seed cultures were then used to inoculate 500 ml bioreactors (Sartorius BioStat-Q Plus, Göttingen, Germany) containing 250 ml of either MSM with 20 g/l fructose or 1.36 g/l sodium formate to an initial OD_600 _= 0.1. Fructose enrichments were carried out as chemostats fed with MSM containing 20 g/l fructose, where the dilution rate was initially set at 0.134 h^−1^ (representing 80% of the growth rate measured during the initial 24-h batch growth phase), and gradually increased to a maximum of 0.248 h^−1^. During the cultivation an 8 M NH_4_OH solution was used to control the pH at 6.7 while serving as an additional nitrogen source. Formic acid enrichments were carried out as pH-stats fed with a mixture of 7.5% w/v formic acid and 54 mM NH_3_ and controlling the pH to 6.7. For all bioreactor cultivations, volumes were held constant at 250 ml using an outlet pump connected to a dip tube set to the correct height, the temperature was maintained at 30°C, and air was sparged into the system at a rate of 300 standard cm³/min. The dissolved oxygen concentration was maintained at 25% with varying stirring rate of the two Rushton impellers. After 40–50 generations, serial dilutions of cultures from each enrichment culture were plated onto LB plates from which isolates were chosen for characterization.

### Isolate evaluation in plate readers

To characterize growth of selected isolates in a plate reader, three biological replicates of each isolate were grown overnight in MSM with 2 g/l fructose or MSM with 100 mM PO_4_ (as above but containing 7.956 g/l K_2_HPO_4_ and 7.394 g/l KH_2_PO_4_) and 4.08 g/l (60 mM) sodium formate supplemented with Gent15. Overnight cultures were diluted to OD_600 _≈ 0.05 in the same medium and grown to mid-log phase (≈20 h). Cultures were then adjusted to OD_600 _= 0.02 in fresh medium without gentamicin. 200 µl of each triplicate culture was transferred to a 100-well plate and incubated in a Bioscreen C Pro microplate reader (Growth Curves USA) at 30°C with continuous, fast orbital shaking at maximum amplitude and absorbance at 600 nm measurements every 15 min. The period of maximum growth was identified over a 4-h window using ordinary least-squares regression from the scikit-learn Python library (sklearn.linear_model.LinearRegression) as the period with the greatest increase in OD_600_ where r^2 ^≥ 0.95. The maximum specific growth rate (μ) was calculated over this period by fitting a polynomial to ln-transformed OD_600_ data using the NumPy Python library (numpy.polyfit). Differences in maximum μ were evaluated using a two-sided, independent *t*-test from the SciPy Python library (scipy.stats.ttest_ind).

### Whole-genome sequencing

To identify genome deletions, selected isolates were subjected to whole-genome nanopore sequencing (Plasmidsaurus). Raw nanopore reads were aligned to *C. necator* H16 reference genome assembly ASM479872v1 (RefSeq GCF_004798725.1, GenBank GCA_004798725.1) using Geneious Prime v2025.1.3 (Dotmatics), and alignments were manually examined to identify deletion boundaries. To identify other mutations in the isolates that may also have contributed to strain fitness, breseq (version 0.38.1) was run on the WGS reads of all isolates, using the nanopore flag. gdtools was then used to aggregate and compare high-confidence mutations across strains. To identify relevant small mutations, the breseq output was filtered to remove (i) all mutations in the parent strain CHC023 or CHC123 that were present in all progeny; (ii) all deletions >200 bp; (iii) all synonymous single nucleotide polymorphisms (SNPs). One mutation with lower confidence in gdtools compare, a 17-unit 7-bp tandem repeat in gene E6A55_08655, was manually confirmed to be present at variable copy number across the different strains and was thus retained in the small mutation analysis. Deletions and other mutations identified in all sequenced isolates are described in Supplementary Tables S1 and S2.

### Isolate evaluation in bioreactors

To evaluate growth of chosen isolates in bioreactors, triplicate seed cultures of each strain were inoculated from LB-Gent15 plates into LB-FN10-Gent15 seed cultures to an OD_600 _≈ 0.05 and incubated for ≈ 14 h. Seed cultures were then centrifuged at 4000 rpm for 10 min, and the cell pellets resuspended in the same bioreactor medium used in the growth enrichment cultivations supplemented with either 40 g/l fructose or 1.36 g/l sodium formate. These seed cultures were then used to inoculate 500 ml bioreactors (Sartorius BioStat-Q Plus, Göttingen, Germany) to an OD_600 _≈ 0.1–0.2. Strains characterized on fructose were cultivated in batch mode using 300 ml bioreactor medium supplemented with 40 g/l fructose. During the cultivation, a 2 M NH_4_OH solution was used to maintain the pH at 6.7. Strains characterized on formic acid were grown under a pH-stat fed-batch mode, with an initial 250 ml bioreactor medium supplemented with 1.36 g/l sodium formate and fed with 200 ml bioreactor medium supplemented with 35% w/v formic acid and 250 mM NH_3_, controlling the pH at 6.7. For all bioreactor cultivations, the temperature was maintained at 30°C, and air was sparged into the system at a rate of 300 standard cm³/min. The dissolved oxygen concentration was maintained at 25% with varying stirring rate of the two Rushton impellers.

## Results

### Generating reduced-genome libraries in *C. necator*

**Figure 1.**
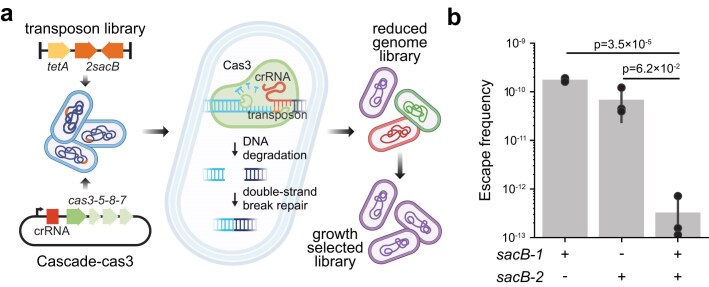
Generating random genomic deletions with TRIM3. (**A**) To generate genome reductions, a transposon carrying selectable (tetracycline resistance, *tetA*) and a pair of counterselectable (sucrose sensitivity, *2sacB*) markers is first randomly inserted into the genome. A plasmid carrying genes for rhamnose-inducible expression of the Cascade–Cas3 complex (*cas3, cas5, cas8*, and *cas7*) and a crRNA targeting the transposon is then introduced into the transposon library. Upon induction, Cas3 nicks the target transposon sequence and degrades adjacent genomic DNA, resulting in large deletions following repair of the double-strand break through endogenous mechanisms. The resulting library of reduced-genome mutants can then be growth selected to isolate mutants with beneficial reductions. (**B**) To evaluate the escape frequency of transposon-library isolates from sucrose selection, serial dilutions of transposon libraries expressing *sacB* from pK18msB [60] (*sacB-1*), *sacB* codon-optimized for *C. necator* (*sacB-2*), or both (2*sacB: sacB-1* + *sacB-2*) were plated on both LB plates and YTS plates containing 15% sucrose. The escape frequency was calculated as the number of colonies on YTS plates divided by the number of colonies on LB plates. Data points represent individual replicates, while bars and error bars represent the average ±1 standard deviation, respectively, of three biological replicates. *P*-values were calculated using a two-sided *t*-test.

To develop a pool of reduced-genome *C. necator* mutants, we devised a strategy for transposon-mediated random iterative minimization with Cascade–Cas3 (TRIM3) (Fig. 1A). First, a nonreplicative plasmid carrying a Tn5 transposase and transposon with selectable and counterselectable markers is introduced into the parent strain. Cells that successfully integrate the transposon into their genome are selected by antibiotic resistance. A replicative plasmid for inducible expression of a Cascade–Cas3 and a crRNA targeting the transposon sequence is then introduced into the transposon library. Once expression is induced, Cascade–Cas3 nicks the transposon sequence and processively degrades adjacent DNA [57, 65]. The resulting double-strand break must be repaired by endogenous processes for the cell to survive. Selecting against cells still carrying the counterselectable marker in the original transposon sequence results in a pool of mutants that have lost the transposon sequence, presumably through deletion of the transposon and adjacent genomic DNA by Cascade–Cas3. This genome-reduced library can then be cultivated under the desired growth conditions to enrich for isolates with improved growth characteristics. Isolates from the enriched population can be evaluated and sequenced using long-read nanopore sequencing to identify beneficial deletions.

We first identified suitable markers to include in the transposon cargo to select for transposon integration and to counterselect against isolates that retain the transposon after genome reduction by Cascade–Cas3. We evaluated growth of a parental *C. necator* in the presence of varying concentrations of antibiotics and observed sensitivity to chloramphenicol, erythromycin, kanamycin, spectinomycin, and tetracycline (Supplementary Fig. S1). As potential selectable markers, we evaluated chloramphenicol and tetracycline resistance genes previously shown to function in *C. necator* [66] using strains in which these antibiotic resistance genes were integrated into the Δ*phaCAB* locus of CHC023 as previously described [55], generating EMF069 (*cat*) and EMF070 (*tetA*). CHC023 is a *C. necator* H16 derivative that includes deletions of a Type I restriction enzyme subunit to improve introduction of heterologous DNA [67] and of the *phaCAB* operon. We chose *tetA*-mediated tetracycline resistance due to strong selection against parental *C. necator* at tetracycline concentrations that did not inhibit growth in a *tetA*-expressing strain (Supplementary Fig. S2). To develop a robust counterselection, we evaluated transposon variations carrying *sacB*, which imposes sensitivity to sucrose and has previously been used for counterselection in *C. necator* [55]. We tested three different *sacB* cassette designs: one expressing a *sacB* sequence from pK18msB [60] (*sacB*-*1*), a second expressing *sacB* we codon-optimized for *C. necator* (*sacB*-*2*), and a third version expressing both (2*sacB*). If the two *sacB* genes in the *2sacB* cassette were placed in the same orientation, it would be possible for recombination between small regions of homology shared by the alleles to delete the intervening region, resulting in a single *sacB* gene that could then be inactivated by a second mutation. While we considered that unlikely, we decided to avoid this possibility by placing the *sacB* genes in the opposite orientation so that recombination between the alleles would result in inversion of the intervening sequence and maintain two *sacB* genes. When we compared the frequency of sucrose-resistant, “escapee” mutants from each of these transposon libraries on sucrose selective plates to the total number of colonies on nonselective plates, we observed a significant decrease (*P *<.05) in the number of escapees from transposon libraries carrying both *sacB* markers (2*sacB*) compared to *sacB-1*, and a large, though not statistically significant (*P* >.05), decrease compared to *sacB-2* (Fig. 1B). We hypothesized that the reduced escape frequency observed with the 2*sacB* cassette was due to a reduced likelihood of mutations inactivating both copies of *sacB*. To test this theory, we sequenced the transposon from escapee isolates of each library. All escapee isolates from the *sacB*-*1* (12/12 isolates) and *sacB*-*2* (11/11 isolates) libraries contained insertions, deletions, or substitutions in *sacB* that putatively inactivate SacB function. Whole-genome sequencing of escapee isolates from the *2sacB* library showed either deletions spanning both copies of *sacB* (2/5 isolates), deletions of the entire transposon and flanking genomic sequences (2/5 isolates), or a wild-type genome with no evidence of the transposon insertion (1/5 isolates). These results suggest that the double *sacB* cassette increases the stringency of the counterselection by insulating against indels and point mutations that can inactivate single copies of *sacB*. Based on these results, we selected the transposon expressing *tetA* and 2*sacB* for generation of a reduced-genome library.

Following the workflow of TRIM3, we next developed a protocol to generate libraries of genome-reduced mutants in *C. necator* (Supplementary Fig. S3). As the parent strain for a genome-reduced library, we chose CHC023. Deletion of the *phaCAB* operon in this strain prevents it from producing polyhydroxybutyrate (PHB), providing a more relevant metabolic context for further engineering to produce other products and preventing accumulation of PHB granules that can influence cell density measurements [55]. First, we generated a transposon library carrying the *tetA* and 2*sacB* markers by conjugating an *E. coli* donor strain carrying a nonreplicative plasmid for expression of the Tn5 transposase and transposon into CHC023. Next-generation sequencing of the library revealed 4357 unique insertions spread relatively evenly across the genome, with a few “hot spots” exhibiting higher transposon densities on chromosome 1 and chromosome 2 (Supplementary Fig. S4). We then performed two serial selections on plates containing tetracycline to encourage propagation of the *tetA*-transposon sequence through every copy of the genome. After tetracycline selection, we introduced a replicative plasmid coding for rhamnose-inducible expression of Cascade–Cas3 and a crRNA targeting the *sacB-1* sequence within the 2*sacB* cassette. After selecting for transconjugants and inducing Cascade–Cas3 expression, we performed two successive selections on sucrose plates to select for isolates that no longer retain SacB activity and putatively contain random genomic deletions. We then pooled the sucrose-resistant isolates to generate a reduced-genome library.

### Selecting isolates with improved growth on formate and fructose

We next enriched the reduced-genome CHC023 library for isolates with improved growth on the heterotrophic substrate fructose or on the C1 substrate formic acid, which can be made electrochemically from CO_2_ and is a potential feedstock for carbon-negative biomanufacturing [29]. *Cupriavidus necator* can grow on formate as the sole source of carbon and energy through activity of formate dehydrogenases that ultimately (re)generate CO_2_ and a reducing equivalent [20, 25]. To enrich for isolates with faster growth in bioreactors, we grew the library with either pH-stat feeding of formic acid or chemostat-mode feeding of fructose. We monitored OD_600_ and volume fed over the course of the run to calculate the number of generations (Supplementary Fig. S5). After 40–50 generations, we picked isolates from each library to evaluate for improved growth on the respective substrates. Following an initial screen to identify candidates with improved growth in a plate reader (see **Supplementary Methods**), we selected five isolates from each selection for further characterization and sequencing (Supplementary Fig. S6).

**Figure 2.**
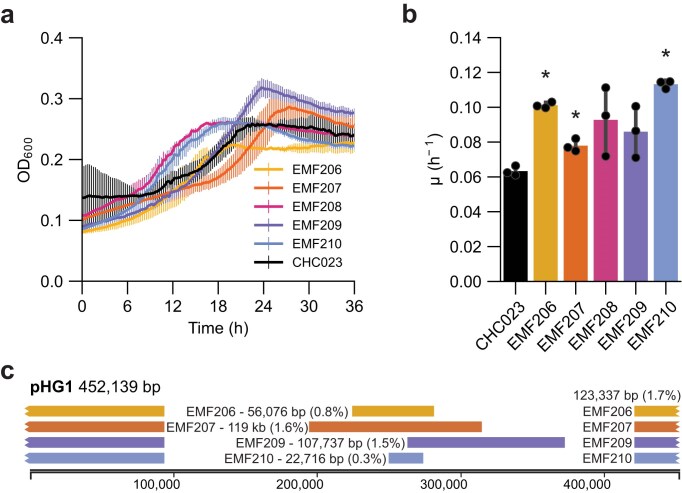
Evaluation of formic acid-selected CHC023 isolates. (**A**) Five isolates from the CHC023 genome-reduced library selected on formate were grown in minimal medium with 4.08 g/l sodium formate as the sole carbon source, along with CHC023, in a plate reader. (**B**) Growth rates of each selected isolate and CHC023 on formate. Three isolates grew significantly faster than CHC023 (*P <.05). (**C**) Map of the megaplasmid (pHG1) showing deletions present in four selected isolates, along with the size of each deletion in bp and as a percentage of the full-length genome. Data represent the average of three biological replicates, ±1 standard deviation. *P*-values were calculated using a two-sided *t*-test.

First, we evaluated the five isolates derived from the CHC023 library enriched during growth on formic acid for improved growth on formate in a plate reader (Fig. 2A) and calculated the maximum growth rate of each isolate (Fig. 2B). Compared to the CHC023 parent strain, 3/5 isolates had a significantly faster maximum specific growth rate (μ). To identify deletions in these strains, we sequenced the genomes of these three isolates plus one additional isolate using long-read nanopore sequencing. All sequenced isolates contained deletions in the megaplasmid ranging from 146 to 242 kb (representing 2.0%–3.3% of the genome) (Fig. 2C and Supplementary Table S1). Each isolate contained an identical 123-kb deletion encompassing the megaplasmid copy of the CBB operon and operons encoding the membrane-bound hydrogenase, regulatory hydrogenase, and soluble hydrogenase, along with a “junkyard region” containing a number of transposons, integrases, and recombinases [23]. Additionally, each isolate contained a second deletion ranging from 22 to 119 kb in a region including genes for denitrification. In isolate EMF209, the deletion extended into a neighboring region containing genes for conjugative transfer of the megaplasmid. Notably, the occurrence of deletions exclusively in the megaplasmid suggests that the hot spots of transposon insertion observed in chromosomes 1 and 2 (Supplementary Fig. S4) did not influence the deletions recovered.

We also examined the genome sequence of each isolate for the presence of other mutations, such as SNPs and/or small indels (<200 bp), that could have arisen during library construction or growth enrichment and contributed to the observed fitness improvements. The genomes of these isolates contained two (EMF207, EMF210), three (EMF206), and nine (EMF209) mutations in addition to the deletions described above (Supplementary Tables S1 and S2). Interestingly, an identical 10 bp deletion in *waaC* (E6A55_14710), which encodes a lipopolysaccharide heptosyltransferase, was present in EMF207, EMF209, and EMF210. The presence of this deletion in multiple isolates suggests it could have occurred in the library prior to the Cascade–Cas3 deletions or could have arisen and been enriched in all three isolates independently. The latter case could suggest that this *waaC* mutation contributed to the improved growth of EMF207, EMF209, and EMF210 on formic acid, although it would be unexpected for an identical mutation to have occurred independently rather than different mutations affecting expression or activity of this enzyme. EMF207 also contained a 12 bp deletion in *phcR* (E6A55_15895). PhcR is thought to be part of a two-component system that represses expression of PhcA, which itself is a transcriptional regulator that we previously found to be important for growth of *C. necator* on formate [55]. Together, these observations suggest that this deletion in *phcR* could benefit growth on formate.

**Figure 3.**
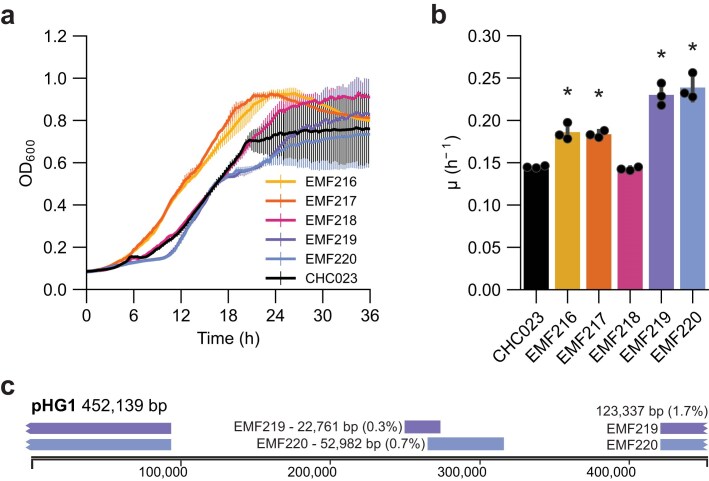
Evaluation of fructose-selected CHC023 isolates. (**A**) Five isolates from the CHC023 genome-reduced library selected on fructose were grown in minimal medium with 2 g/l fructose as the sole carbon source, along with CHC023, in a plate reader. (**B**) Growth rates of each selected isolate compared to the growth rate of CHC023 on fructose. Four isolates grew significantly faster than CHC023 (*P <.05). (**C**) Map of the megaplasmid (pHG1) showing deletions present in two selected isolates, along with the size of each deletion in bp and as a percentage of the full-length genome. Data represent the average of three biological replicates, ±1 standard deviation. *P*-values were calculated using a two-sided *t*-test.

We next evaluated five isolates derived from the population enriched by growth on fructose in a plate reader (Fig. 3A) and compared the maximum growth rate to that of CHC023 (Fig. 3B). Four of the five isolates exhibited significantly faster maximum growth rates (*P* <.05) compared to the parent strain. Whole-genome sequencing of two isolates, EMF219 and EMF220, also revealed the same 10 bp deletion in *waaC* identified in the formate-derived isolates EMF207, EMF209, and EMF210 and the same 12 bp deletion in *phcR* that was observed in the formate-derived isolate EMF207, supporting a general fitness benefit for these mutations given that they appear to have arisen in both formate and fructose growth enrichments. The fructose-selected isolates also contained deletions in the megaplasmid totaling 146–176 kb (2.0%–2.4% of the genome), including a 123-kb deletion identical to the deletion observed in the formic acid-selected isolates and a second deletion encompassing denitrification operons (Fig. 3C). The presence of similar deletions in isolates from formate and fructose selections suggests a strong selective pressure for deleting the CBB and/or hydrogenase operons, aligning with results from a previous ALE on formate which resulted in hydrogenase and CBB operon deletions [55]. This selective pressure may result from the high proteomic burden of hydrogenase expression in *C. necator* H16 [54], where a mutation in the regulatory hydrogenase hinders its ability to suppress hydrogenase expression in the absence of H_2_ [68]. Deletion of the megaplasmid CBB operon copy has been reported to increase expression of the chromosome 2 CBB operon [55]. This likely explains why this deletion is advantageous during growth on formate, which relies on the CBB cycle. Because the megaplasmid is dispensable during growth on many substrates [55], we hypothesized that generating and selecting reduced-genome libraries that lack the megaplasmid may help identify novel chromosomal deletions that improve growth on fructose and formate.

### Identifying beneficial chromosomal deletions on formate and fructose

To identify genome reductions outside of the megaplasmid that lead to improved growth on fructose and formate, we applied TRIM3 to CHC123, a CHC023 derivative with the megaplasmid deleted [55]. Prior to introducing Cascade–Cas3, we found that the transposon library contained 5054 unique insertions spread across the genome with a remarkably similar occurrence of hot spots to those observed in the CHC023 library (Supplementary Fig. S4). As before, we grew the reduced-genome CHC123 library on formic acid or fructose in bioreactors, monitored growth (OD_600_) and volume fed, and calculated the number of generations that had occurred (Supplementary Fig. S7). After 40–50 generations, we selected isolates from each enriched library and performed an initial growth evaluation to identify faster-growing mutants (Supplementary Fig. S8).

**Figure 4.**
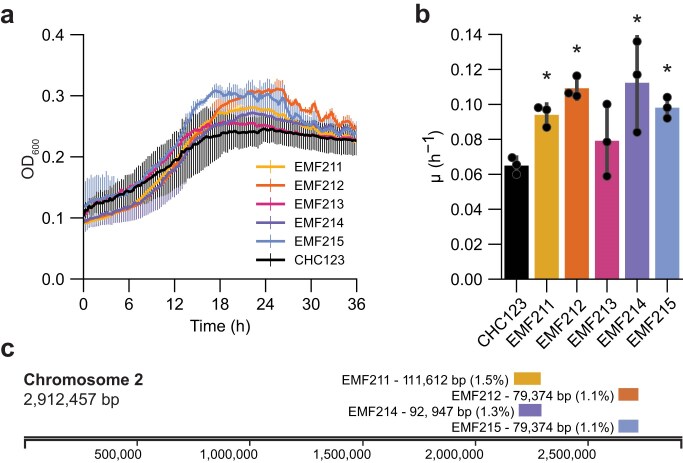
Evaluation of formic acid-selected CHC123 isolates. (**A**) Five isolates from the CHC123 genome-reduced library selected on formic acid, along with CHC123, were grown in minimal medium with 4.08 g/l sodium formate as the sole carbon source in a plate reader. (**B**) Growth rates of each selected isolate compared to the growth rate of CHC123 on formate. Four isolates grew significantly faster than CHC123 (*P <.05). (**C**) Map of chromosome 2 showing deletions present in four selected isolates, along with the size of each deletion in bp and as a percentage of the full-length genome (including the megaplasmid). Data represent the average of three biological replicates, ±1 standard deviation. *P*-values were calculated using a two-sided *t*-test.

We first characterized five promising isolates from the formic acid-enriched library for growth on formate in a plate reader (Fig. 4A) and identified four isolates whose maximum growth rates were significantly improved compared to CHC123 (Fig. 4B). Following whole-genome sequencing, we identified single deletions in chromosome 2 in each isolate, ranging from 79 to 111 kb (1.1%–1.5% of the full-length genome including the megaplasmid; Fig. 4C). Isolates EMF211 and EMF214 contained overlapping, but not identical, deletions in a region of chromosome 2 that contains genes associated with secondary metabolism. Isolates EMF212 and EMF215 contained identical deletions in a different region of chromosome 2 that has genes associated with secondary metabolism as well as the *fli* operon required for flagella biosynthesis. EMF214 contained a 3 bp insertion in *ptsP* (E6A55_01660), which encodes a component of a phosphoenolpyruvate:carbohydrate phosphotransferase system. EMF206 contained a different mutation in *ptsP*, suggesting it might be important for formate catabolism (Supplementary Table S1). EMF211 and EMF215 contained 54 and 93 bp deletions, respectively, in E6A55_03500, a gene predicted to encode an ATP binding protein of unknown function (Supplementary Table S1). While it is unknown if these smaller deletions were induced by TRIM3 or arose spontaneously, the formic acid-selected CHC023-derived isolate EMF209 also had an L85P mutation in E6A55_03500, suggesting that these mutations contribute to improved growth on formate.

**Figure 5.**
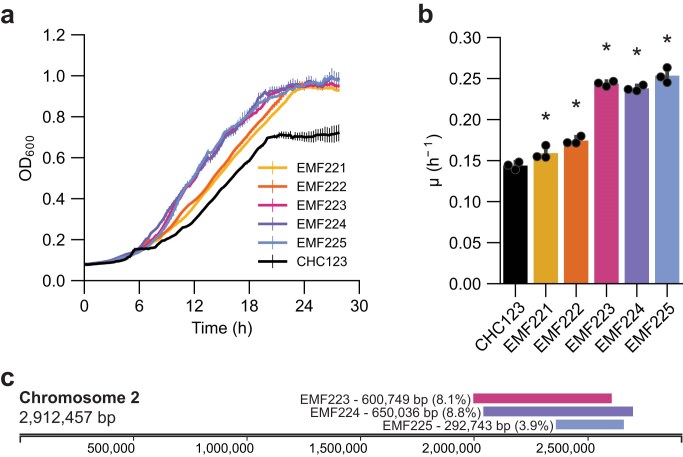
Evaluation of fructose-selected CHC123 isolates. (**A**) Five isolates from the CHC123 genome-reduced library selected on fructose were grown in minimal medium with 2 g/l fructose as the sole carbon source along with CHC123 in a plate reader. (**B**) Growth rates of each selected isolate compared to the growth rate of CHC123 on fructose. All five isolates grew significantly faster than CHC123 (*P <.05). (**C**) Map of chromosome 2 showing deletions present in three selected isolates, along with the size of each deletion in bp and as a percentage of the full-length genome. Data represent the average of three biological replicates, ±1 standard deviation. *P-*values were calculated using a two-sided *t*-test.

We similarly characterized five promising isolates from the fructose-selected CHC123 library (Fig. 5A), all of which grew significantly (*P *<.05) faster than CHC123 in a plate reader (Fig. 5B). The three fastest growing isolates (EMF223, EMF224, and EMF225) had 293–650 kb (3.9%–8.8% of the genome) deletions in the same region of chromosome 2 (Fig. 5C) containing secondary metabolism and denitrification genes (e.g. *narH*). The deletion in isolate EMF224 also spanned the *fli* operon. Similar to our observations using the CHC023 library, the deletions in chromosome 2 recovered following selection of the CHC123 library on formic acid and fructose did not occur near the few areas of greatest transposon insertion density (Supplementary Fig. S4), suggesting these hot spots did not skew their selection. EMF223 and EMF225 also contained the same 12 bp deletion in *phcR* that we observed in other formate and fructose selected isolates (Supplementary Table S1).

**Figure 6.**
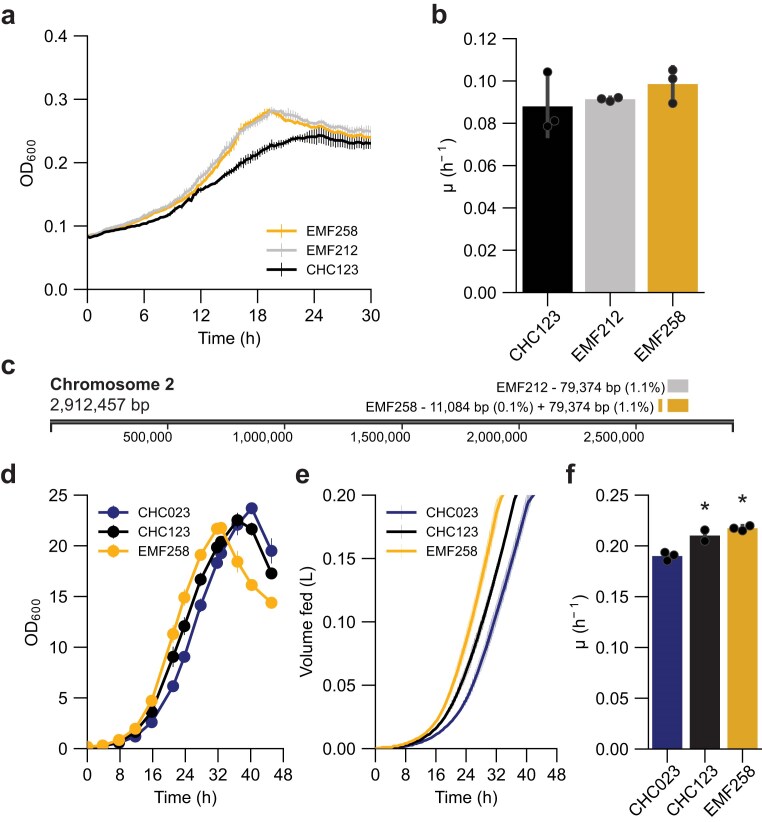
Evaluation of second-generation formic acid-selected isolates. (**A**) One isolate from the second-generation reduced genome library derived from EMF212 selected on formic acid was grown in minimal medium with 4.08 g/l sodium formate as the sole carbon source along with CHC123 and EMF212 in a plate reader. (**B**) Growth rates of both strains compared to the growth rate of CHC123 in a plate reader. Neither grew significantly faster than CHC123 (*P *>.05). (**C**) Map of chromosome 2 showing deletions present in Isolate EMF258 and the parent strain EMF212, along with the size of each deletion in bp and as a percentage of the CHC023 genome. (**D**) OD_600_ measurements and (**E**) volume fed for strains grown in pH-stat bioreactors fed 200 ml of 35% w/v formic acid. (**F**) Maximum growth rates of CHC023, CHC123, and isolate EMF258 in bioreactors. CHC123 and EMF258 grew significantly faster than CHC023 (*P <.05). Except for duplicates for CHC123 in panels (D)–(F), data represent the average of three biological replicates, ±1 standard deviation. For CHC123 in panels (D)–(F), data represents the average of two biological replicates, ±1 standard deviation. *P*-values were calculated using a two-sided *t*-test.

Given the increase in growth rate following one round of genome reduction and selection, we hypothesized that iteratively performing TRIM3 may yield further improvements. To demonstrate this, a second round of TRIM3 was applied to formate-selected CHC123 isolate EMF212 (Fig. 4), one of the isolates containing deletions of the *fli* operon and secondary metabolism operons. Following enrichment on formic acid in a bioreactor (Supplementary Fig. S9), we characterized growth of an isolate in a plate reader relative to its parent, EMF212, as well as the parent of EMF212, CHC123 (Fig. 6A). Isolate EMF258 showed a modest, though not statistically significant, improvement in growth rate under these conditions (Fig. 6B). Sequencing of this isolate revealed a K98T mutation in *waaC* (Supplementary Table S1) and an additional 11 kb deletion on chromosome 2, encompassing genes associated with secondary metabolism (Fig. 6C). In addition to the 79 kb deletion on chromosome 2 inherited from EMF212, this strain also inherited the deletion of the 452-kb megaplasmid from CHC123, resulting in a total reduction of 542 kb (7.3% of the genome) compared to CHC023. We next sought to compare growth of these strains in bioreactors under fed-batch mode using pH-stat feeding of formic acid (Fig. 6D), conditions that are more relevant to industrial processes compared to the plate reader growths used to screen isolates. Under these conditions, isolate EMF258 consumed formic acid faster than either parent strain (Fig. 6E) and exhibited a 10% faster growth rate than CHC123 and a 14% faster growth rate than CHC023 (Fig. 6F).

**Figure 7.**
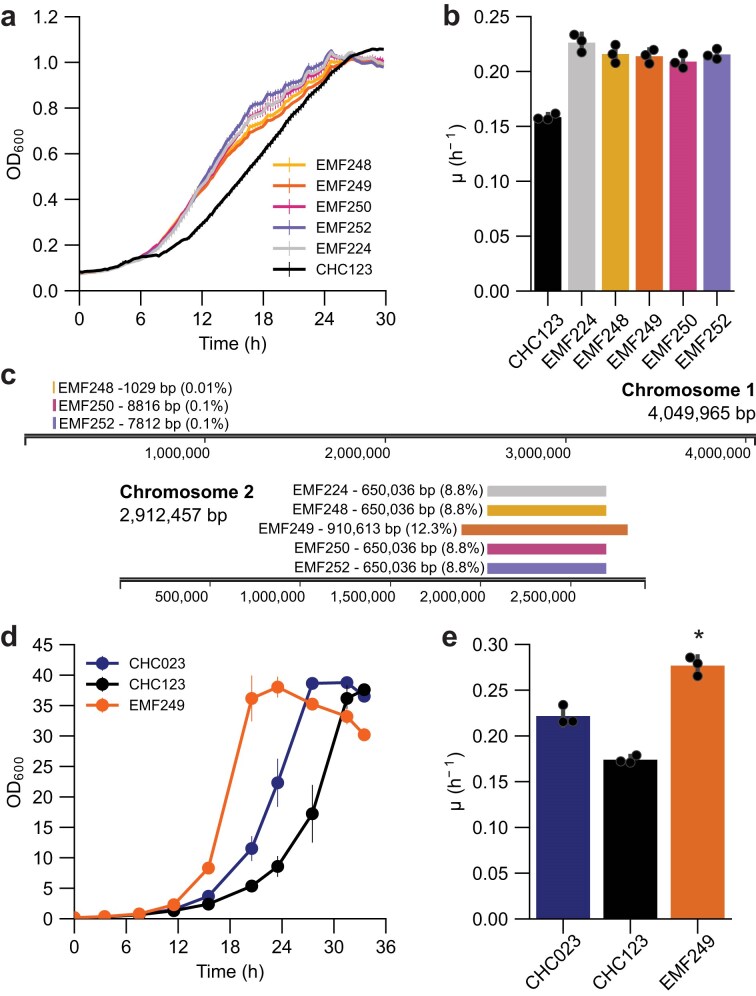
Evaluation of second-generation fructose-selected isolates. (**A**) Four isolates from the second-generation reduced-genome library derived from EMF224 selected on fructose were grown in minimal medium with 2 g/l fructose as the sole carbon source along with CHC123 and EMF224 in a plate reader. (**B**) Growth rates of all strains compared to the growth rate of EMF224 in a plate reader. No isolates grew significantly faster than EMF224 (*P *>.05). (**C**) Maps of chromosomes 1 and 2 showing deletions present in the selected isolates and the parent strain EMF224, along with the size of each deletion in bp and as a percentage of the full-length genome. (**D**) OD_600_ measurements and (**E**) maximum growth rates of CHC023, CHC123, and isolate EMF249 grown on fructose in a bioreactor. EMF249 grew significantly faster than CHC023 (*P <.05). Data represent the average of three biological replicates, ±1 standard deviation. *P-*values were calculated using a two-sided *t*-test.

To further assess the benefits of multiple rounds of TRIM3 on growth optimization, we also performed a second round of TRIM3 on fructose-selected CHC123 isolate EMF224 (Fig. 5), which contained a 650 kb deletion on chromosome 2. Following preliminary isolate screening (Supplementary Fig. S10), we characterized four second-generation isolates in a plate reader compared to their parent EMF224, and the parent of EMF224, CHC123 (Fig. 7A). These isolates exhibited similar growth rates to EMF224 (Fig. 7B). Sequencing revealed that all four isolates contained different nonsynonymous mutations in E6A55_01580, a GntR family transcriptional regulator gene (Supplementary Table S1). The occurrence of these mutations only in EMF224-derived isolates might suggest an interaction with the large deletion it contains in chromosome 2. Isolates EMF248, EMF250, and EMF252 had 1–9 kb deletions in chromosome 1 that include a transcriptional regulator gene of unknown function and genes for methionine biosynthesis (metH; Fig. 7C) in addition to the 650 kb deletion in chromosome 2 inherited from EMF224. Isolate EMF249 had an additional 261 kb deletion in chromosome 2 that extended the 650 kb deletion from EMF224 to 910 kb, encompassing additional secondary metabolism operons and resulting in a total deletion of 1.36 Mb when including the initial 452 kb megaplasmid deletion (18.4% of the wild-type genome). Because isolate EMF249 represented the largest genome reduction, we chose this isolate to compare to CHC023 and CHC123 in bioreactors containing fructose under batch mode (Fig. 7D). Despite having previously observed more rapid growth of CHC123 relative to CHC023 on fructose in a plate reader [55], CHC023 outperformed CHC123 in this bioreactor experiment, perhaps due to the differences in these conditions (e.g. aeration, pH control). EMF249, however, exhibited a 25% faster growth rate than CHC023 (*P *<.05; Fig. 7E).

## Discussion

In this work, we developed a method for bacterial genome reduction, TRIM3, and demonstrated its utility in the industrial host *C. necator* H16 by selecting and characterizing isolates with large deletions that improve growth on fructose and formate. TRIM3 harnesses recent advances in Cascade–Cas3 editing, which can generate large genomic deletions through the processive nuclease activity of Cas3 [57], and in long-read DNA sequencing, which enables easy mapping of deletions without DNA barcoding. To reduce the abundance of mutants capable of escaping sucrose counterselection, we generated a gene cassette containing two *sacB* genes, a strategy that could be helpful in other methods in which inactivating mutations might occur at a similar frequency as the desired excision or recombination. By combining these advances with transposon mutagenesis and growth-coupled selection, TRIM3 enables iterative, random genome reduction and facile selection of improved strains. While some rational genome reduction approaches have proven fruitful in generating genome-reduced strains with improved phenotypes [3–13], the process is generally labor-intensive, often requiring many serial deletions to generate a strain with a substantially reduced genome. Moreover, knowledge of the host’s genome, metabolism, and physiology in the context of the conditions of interest is often incomplete, such that rationally genome-reduced strains may not perform better in those conditions after all. TRIM3 overcomes these challenges by creating a library of cells with random deletions of variable sizes (ranging from 11 to 650 kb in this study), enriching this library for those with improved growth in the conditions of interest, and identifying deletions in selected isolates by long-read sequencing. We demonstrated two rounds of TRIM3 genome reduction, but the process could be repeated until no further deletions exist that improve performance and, thereby, enable selection. This iterative strategy should be applicable to a multitude of bioprocessing conditions and chassis, providing a versatile platform for generating improved industrial hosts.

With a genome notable for its large size and genetic redundancy [53], *C. necator* H16 was an excellent candidate for genome reduction using TRIM3. Using TRIM3, we reduced the size of the genome from 7.4 to 6.1 Mb, an 18.4% genome reduction that results in 25% faster growth on fructose relative to the near-wild-type parental strain, CHC023 (Fig. 7E). We also generated a strain with a total genome reduction of 0.5 Mb (7.3% of the genome) that grows 14% faster on formate, a C1 substrate of potential industrial interest [29]. *Cupriavidus necator*’s metabolic versatility also suggests that TRIM3 could be applied to identify growth-improved strains on a variety of other industrially relevant substrates and could be coupled with additional metabolic engineering strategies. For example, introducing a point mutation in the gene encoding the PTS transporter NagE while also deleting the gene encoding the transcriptional repressor NagR has been shown to enable growth of *C. necator* H16 on the more affordable and industrially relevant substrate glucose [22, 42], which could be combined with the genome reductions identified in this study or with a new TRIM3-generated library selected on glucose. It would be a worthy pursuit of future work to engineer the strains generated here to produce targeted products, as we expect their improved growth rates could translate to improved productivity. Some rationally genome-reduced strains have not outperformed their parent strains in producing targeted products [16], and this pitfall may also apply in TRIM3-generated strains if genes that are dispensable for growth are still important for production. Coupling randomly reduced strains with synthetic biology approaches that make growth and/or survival reliant on production [69–71] may help circumvent this issue and perhaps even be leveraged to improve production.

The goal of our study was to develop and demonstrate TRIM3 as a tool to improve performance of a bioprocessing host in defined conditions, but the deletions generated using TRIM3 could also provide valuable insights into a host’s genome, metabolism, and physiology. And while further study leveraging complementation and epistasis analysis would be required to define genotype–phenotype relationships, broad patterns emerge from the mutations and deletions generated in this study. Convergent mutations in *waaC, phcR, ptsP*, E6A55_03500, and E6A55_01580 (Supplementary Table S1) suggest that they might contribute to the improved growth rates exhibited by the TRIM3 isolates. Conversely, a previous transposon mutagenesis study in *C. necator* explored the fitness impact of different gene disruptions under various growth conditions, including continuous growth on fructose and formate. None of the convergent mutations in our isolates were significantly enriched or depleted during growth on formate and only mutations in *phcR* were previously found to be beneficial for growth on fructose [54]. This suggests that, with the exception of *phcR* on fructose, these mutations may confer environment-specific benefits unrelated to carbon source, that they may provide growth benefits in tandem with the large deletions observed in this study, or may confer smaller fitness effects that take longer to arise in the population. The consistent deletion of large portions of the pHG1 megaplasmid when TRIM3 was applied to CHC023 on both formate and fructose reflects a growth benefit previously observed when pHG1 was partially or entirely deleted [55, 56] and the finding that the CBB and hydrogenase operons on the megaplasmid are expressed unnecessarily and represent a biological burden under nonautotrophic growth conditions [54]. In a previous study in which we performed ALE of *C. necator* H16 grown on formate [55], we observed large deletions in the pHG1 megaplasmid spanning the CBB operon and hydrogenases that were nearly identical to the 123-kb deletion observed in CHC023-derived isolates in the present study, suggesting strong selection for this deletion. Coupled with the observation that all CHC023-derived TRIM3 isolates exhibited two deletions, it is likely that the 123-kb deletion they share occurred spontaneously, perhaps even during library construction. This deletion is flanked at the 3′ end by an ISAe1 family transposase found in both the megaplasmid and chromosome 1, and immediately internal to the deletion is an integrase, suggesting that this deletion could have resulted from mobile genetic element activity. When TRIM3 was subsequently applied to CHC123, in which the megaplasmid was already deleted, we frequently observed deletions in a 910 kb region of chromosome 2 (1,902,070–2,812,682), consistent with the assertion that chromosome 2 primarily includes secondary metabolic functions while chromosome 1 encodes more essential housekeeping functions and up to 80% of the proteome by mass [20, 52]. This region includes genes associated with catabolism of alternative carbon sources (notably, the *fdw* formate dehydrogenase operon that appears to be less important for formate oxidation than other formate dehydrogenases expressed by *C. necator* [54]), anabolism of secondary metabolites, and functions that are unnecessary and perhaps even burdensome in the controlled environment of a bioreactor such as chemotaxis (*che*), copper resistance, toxin-antitoxin systems, secretion (*tss*), motility (*motAB*), and flagella biosynthesis (*fli)*, whose deletion has been shown to improve growth on fructose in *Pseudomonas putida* KT2440 [72]. The deletions in this region identified in the fructose-selected CHC123 isolates (Fig. 5) were considerably larger than in the formate-selected CHC123 isolates (Fig. 4) despite having been derived from the same TRIM3 library, suggesting that some of the genes it contains might be important for growth on formate. Interestingly, the second-generation fructose-selected isolate EMF249 (Fig. 7) extended the 650 kb deletion it inherited from its first-generation parent, CHC123 TRIM3 fructose isolate EMF224 (Fig. 5), by an additional 261 kb, underscoring the value of performing TRIM3 iteratively. Although the extension of this deletion did not appear to further improve the growth rate in the conditions tested, we consider even neutral deletions to be desirable since a smaller genome should be less prone to spontaneous recombination and off-target edits during genome engineering. Notably, the original 650 kb deletion is, to our knowledge, the largest Cas3-mediated deletion described in bacteria, the next largest being a 424 kb deletion generated in *Pseudomonas aeruginosa* [57]. We expect the size of deletions generated using TRIM3 to vary from host to host based on factors such as the expression and activity of the exogenous transposon and Cascade–Cas3 machinery, the activity of the native DNA repair machinery, and the number and arrangement of essential genes in the genome.

In addition to information about the fitness benefits of deletions, TRIM3 may help illuminate native DNA repair mechanisms. In *C. necator*, homologous recombination has been shown to be the dominant double-strand break repair mechanism for Cas9-induced deletions, whereas nonhomologous end joining is not present or inactive [67]. We examined the sequences near the large deletion junctions in isolates throughout this study and observed small, 2–5 bp microhomologies flanking the junction site in 15 out of 16 unique deletions, while the remaining deletion had no homologous bases (Supplementary Table S3). We hypothesize that strong selective pressure to repair the excision might extend mismatch tolerance for the homologies used for repair, and/or that homologous recombination is complemented by additional repair mechanisms in this strain. Generation and sequencing of additional deletion junctions, as well as closer examination of native protein sequences, could help elucidate DNA repair in both *C. necator* and in other organisms.

Beyond this study, we envision that TRIM3 could be leveraged toward streamlining additional hosts for a wide variety of conditions and generating large-scale genome modifications to help gain a deeper understanding of genotype–phenotype relationships. The plasmids described here should be useful without modification to perform TRIM3 in other transformable Gram-negative bacteria. With appropriate modifications to enable transformation, plasmid replication, gene expression, and selectable and counterselectable markers, constructs developed in this study should also enable TRIM3 to be deployed in Gram-positive bacteria, archaea, and/or eukaryotic microbes. TRIM3 could be performed to improve production of targeted products in strains engineered to impose growth-coupled biosynthesis. In addition to enabling development of improved bioprocessing hosts, TRIM3 could be used to study the effect of multiple gene knockouts on host phenotype. Single-gene knockout libraries have proven useful in elucidating the importance of individual genes during growth in specific conditions in *C. necator* [54, 73] and other organisms, but are limited in their ability to probe combinations of knockouts. Generating TRIM3 libraries with greater coverage and applying deep sequencing of populations might enable observation of how the enrichment of different deletions occurs under varying conditions. While the combination of first-round knockouts would be constrained by their genomic proximity (i.e. their likelihood to be within the same deletion), iterative rounds of TRIM3 might enable deeper analysis of multiplex knockouts. If paired with analysis of single-gene knockout libraries enriched in the same conditions and/or comparisons between multiple conditions, such data could be used to train artificial intelligence and machine learning models capable of deciphering complex data sets to yield novel insights. Overall, we expect that TRIM3 will be a useful tool for generating streamlined, growth-improved bioprocessing hosts and understanding genotype–phenotype relationships.

## Supplementary Material

gkag753_Supplemental_Files

## Data Availability

Sequencing data were submitted to the NCBI Sequence Read Archive (SRA) as BioProject PRJNA1433156.

## References

[1] LeBlanc N, Charles TC. Bacterial genome reductions: tools, applications, and challenges. Front Genome Ed. 2022;4:957289. 10.3389/fgeed.2022.957289 36120530 PMC9473318

[2] Kim K, Choe D, Cho S et al. Reduction-to-synthesis: the dominant approach to genome-scale synthetic biology. Trends Biotechnol. 2024;42:1048–63. 10.1016/j.tibtech.2024.02.008 38423803

[3] Komatsu M, Komatsu K, Koiwai H et al. Engineered *Streptomyces avermitilis* host for heterologous expression of biosynthetic gene cluster for secondary metabolites. ACS Synth Biol. 2013;2:384–96. 10.1021/sb3001003 23654282 PMC3932656

[4] Wynands B, Otto M, Runge N et al. Streamlined *Pseudomonas taiwanensis* VLB120 chassis strains with improved bioprocess features. ACS Synth Biol. 2019;8:2036–50. 10.1021/acssynbio.9b00108 31465206

[5] Zhang F, Huo K, Song X et al. Engineering of a genome-reduced strain *Bacillus amyloliquefaciens* for enhancing surfactin production. Microb Cell Fact. 2020;19:223. 10.1186/s12934-020-01485-z 33287813 PMC7720510

[6] Liu H, Chen Y, Zhang Y et al. Enhanced production of polyhydroxyalkanoates in *Pseudomonas putida* KT2440 by a combination of genome streamlining and promoter engineering. Int J Biol Macromol. 2022;209:117–24. 10.1016/j.ijbiomac.2022.04.004 35395277

[7] Li Y, Zhu X, Zhang X et al. Characterization of genome-reduced *Bacillus subtilis* strains and their application for the production of guanosine and thymidine. Microb Cell Fact. 2016;15:94. 10.1186/s12934-016-0494-7 27260256 PMC4893254

[8] Ahmed Y, Rebets Y, Estévez MR et al. Engineering of *Streptomyces lividans* for heterologous expression of secondary metabolite gene clusters. Microb Cell Fact. 2020;19:5. 10.1186/s12934-020-1277-8 31918711 PMC6950998

[9] Bu Q-T, Yu P, Wang J et al. Rational construction of genome-reduced and high-efficient industrial Streptomyces chassis based on multiple comparative genomic approaches. Microb Cell Fact. 2019;18:16. 10.1186/s12934-019-1055-7 30691531 PMC6348691

[10] Fan X, Zhang Y, Zhao F et al. Genome reduction enhances production of polyhydroxyalkanoate and alginate oligosaccharide in *Pseudomonas mendocina*. Int J Biol Macromol. 2020;163:2023–31. 10.1016/j.ijbiomac.2020.09.067 32941898

[11] Liu J, Zhou H, Yang Z et al. Rational construction of genome-reduced *Burkholderiales chassis* facilitates efficient heterologous production of natural products from proteobacteria. Nat Commun. 2021;12:4347. 10.1038/s41467-021-24645-0 34301933 PMC8302735

[12] Kohlstedt M, Weimer A, Weiland F et al. Biobased PET from lignin using an engineered cis,cis-muconate-producing *Pseudomonas putida* strain with superior robustness, energy and redox properties. Metab Eng. 2022;72:337–52. 10.1016/j.ymben.2022.05.001 35545205

[13] Xu T, Chen J, Mitra R et al. Deficiency of exopolysaccharides and O-antigen makes *Halomonas bluephagenesis* self-flocculating and amenable to electrotransformation. Commun Biol. 2022;5:623. 10.1038/s42003-022-03570-y 35750760 PMC9232590

[14] Karcagi I, Draskovits G, Umenhoffer K et al. Indispensability of horizontally transferred genes and its impact on bacterial genome streamlining. Mol Biol Evol. 2016;33:1257–69. 10.1093/molbev/msw009 26769030 PMC5854090

[15] Reuß DR, Altenbuchner J, Mäder U et al. Large-scale reduction of the *Bacillus subtilis* genome: consequences for the transcriptional network, resource allocation, and metabolism. Genome Res. 2017;27:289–99. 10.1101/gr.215293.116 27965289 PMC5287234

[16] Amendola CR, Cordell WT, Kneucker CM et al. Comparison of wild-type KT2440 and genome-reduced EM42 *Pseudomonas putida* strains for muconate production from aromatic compounds and glucose. Metab Eng. 2024;81:88–99. 10.1016/j.ymben.2023.11.004 38000549

[17] Vernyik V, Karcagi I, Tímar E et al. Exploring the fitness benefits of genome reduction in *Escherichia coli* by a selection-driven approach. Sci Rep. 2020;10:7345. 10.1038/s41598-020-64074-5 32355292 PMC7193553

[18] Shaw D, Miravet-Verde S, Piñero-Lambea C et al. LoxTnSeq: random transposon insertions combined with cre/lox recombination and counterselection to generate large random genome reductions. Microb Biotechnol. 2021;14:2403–19. 10.1111/1751-7915.13714 33325626 PMC8601177

[19] Ma S, Su T, Liu J et al. Reduction of the bacterial genome by transposon-mediated random deletion. ACS Synth Biol. 2022;11:668–77. 10.1021/acssynbio.1c00353 35104106

[20] Pohlmann A, Fricke WF, Reinecke F et al. Genome sequence of the bioplastic-producing “Knallgas” bacterium *Ralstonia eutropha* H16. Nat Biotechnol. 2006;24:1257–62. 10.1038/nbt1244 16964242

[21] Schwartz E, Voigt B, Zühlke D et al. A proteomic view of the facultatively chemolithoautotrophic lifestyle of *Ralstonia eutropha* H16. Proteomics. 2009;9:5132–42. 10.1002/pmic.200900333 19798673

[22] Volodina E, Raberg M, Steinbüchel A. Engineering the heterotrophic carbon sources utilization range of *Ralstonia eutropha* H16 for applications in biotechnology. Crit Rev Biotechnol. 2015;36:978–91. 10.3109/07388551.2015.1079698 26329669

[23] Schwartz E, Henne A, Cramm R et al. Complete nucleotide sequence of pHG1: a *Ralstonia eutropha* H16 megaplasmid encoding key enzymes of H2-based lithoautotrophy and anaerobiosis. J Mol Biol. 2003;332:369–83. 10.1016/S0022-2836(03)00894-5 12948488

[24] Friedrich CG, Bowien B, Friedrich B. Formate and oxalate metabolism in *Alcaligenes eutrophus*. J Gen Microbiol. 1979;115:185–92. 10.1099/00221287-115-1-185

[25] Friedrich CG, Friedrich B, Bowien B. Formation of enzymes of autotrophic metabolism during heterotrophic growth of *Alcaligenes eutrophus*. Microbiology. 1981;122:69–78. 10.1099/00221287-122-1-69

[26] Holmes EC, Bleem AC, Johnson CW et al. Adaptive laboratory evolution and metabolic engineering of *Cupriavidus necator* for improved catabolism of volatile fatty acids. Metab Eng. 2024;86:262–73. 10.1016/j.ymben.2024.10.011 39490669

[27] Johnson BF, Stanier RY. Dissimilation of aromatic compounds by *Alcaligenes eutrophus*. J Bacteriol. 1971;107:468–75. 10.1128/jb.107.2.468-475.1971 5113598 PMC246948

[28] Wang W, Yang S, Hunsinger GB et al. Connecting lignin-degradation pathway with pre-treatment inhibitor sensitivity of *Cupriavidus necator*. Front Microbiol. 2014;5:247. 10.3389/fmicb.2014.00247 24904560 PMC4034039

[29] Yishai O, Lindner SN, de la Cruz JG et al. The formate bio-economy. Curr Opin Chem Biol. 2016;35:1–9. 10.1016/j.cbpa.2016.07.005 27459678

[30] Byrom D . Polymer synthesis by microorganisms: technology and economics. Trends Biotechnol. 1987;5:246–50. 10.1016/0167-7799(87)90100-4

[31] Byrom D . Production of poly-β-hydroxybutyrate: poly-β-hydroxyvalerate copolymers. FEMS Microbiol Lett. 1992;103:247–50. 10.1111/j.1574-6968.1992.tb05844.x

[32] Kourmentza C, Plácido J, Venetsaneas N et al. Recent advances and challenges towards sustainable polyhydroxyalkanoate (PHA) production. Bioeng. 2017;4:55. 10.3390/bioengineering4020055 PMC5590474 28952534

[33] Windhorst C, Gescher J. Efficient biochemical production of acetoin from carbon dioxide using *Cupriavidus necator* H16. Biotechnol Biofuels. 2019;12:163. 10.1186/s13068-019-1512-x 31297151 PMC6598341

[34] Krieg T, Sydow A, Faust S et al. CO_2_ to terpenes: autotrophic and electroautotrophic α-humulene production with *Cupriavidus necator*. Angew Chem Int Ed. 2018;57:1879–82. 10.1002/anie.201711302 29232490

[35] Wu H, Pan H, Li Z et al. Efficient production of lycopene from CO_2_ via microbial electrosynthesis. Chem Eng J. 2022;430:132943. 10.1016/j.cej.2021.132943

[36] Jang Y, Lee YJ, Gong G et al. Carbon dioxide valorization into resveratrol via lithoautotrophic fermentation using engineered *Cupriavidus necator* H16. Microb Cell Fact. 2024;23:122. 10.1186/s12934-024-02398-x 38678199 PMC11055273

[37] Crépin L, Lombard E, Guillouet SE. Metabolic engineering of *Cupriavidus necator* for heterotrophic and autotrophic alka(e)ne production. Metab Eng. 2016;37:92–101. 10.1016/j.ymben.2016.05.002 27212691

[38] Gascoyne JL, Bommareddy RR, Heeb S et al. Engineering *Cupriavidus necator* H16 for the autotrophic production of (R)-1,3-butanediol. Metab Eng. 2021;67:262–76. 10.1016/j.ymben.2021.06.010 34224897 PMC8449065

[39] Mishra S, Perkovich PM, Mitchell WP et al. Expanding the synthetic biology toolbox of *Cupriavidus necator* for establishing fatty acid production. J Ind Microbiol Biotechnol. 2024;51:kuae008. 10.1093/jimb/kuae008 38366943 PMC10926325

[40] Chen JS, Colón B, Dusel B et al. Production of fatty acids in *Ralstonia eutropha* H16 by engineering β-oxidation and carbon storage. PeerJ. 2015;3:e1468. 10.7717/peerj.1468 26664804 PMC4675107

[41] Collas F, Dronsella BB, Kubis A et al. Engineering the biological conversion of formate into crotonate in *Cupriavidus necator*. Metab Eng. 2023;79:49–65. 10.1016/j.ymben.2023.06.015 37414134

[42] Wang L, Luo H, Yao B et al. Optimizing hexose utilization pathways of *Cupriavidus necator* for improving growth and L-alanine production under heterotrophic and autotrophic conditions. Int J Mol Sci. 2024;25:548. 10.3390/ijms25010548 PMC10778655 38203719

[43] Wang L, Yao J, Tu T et al. Heterotrophic and autotrophic production of L-isoleucine and L-valine by engineered *Cupriavidus necator* H16. Bioresour Technol. 2024;398:130538. 10.1016/j.biortech.2024.130538 38452952

[44] Wang X, Luo H, Wang Y et al. Direct conversion of carbon dioxide to glucose using metabolically engineered *Cupriavidus necator*. Bioresour Technol. 2022;362:127806. 10.1016/j.biortech.2022.127806 36031135

[45] Löwe H, Beentjes M, Pflüger-Grau K et al. Trehalose production by *Cupriavidus necator* from CO_2_ and hydrogen gas. Bioresour Technol. 2021;319:124169. 10.1016/j.biortech.2020.124169 33254445

[46] Nangle SN, Ziesack M, Buckley S et al. Valorization of CO_2_ through lithoautotrophic production of sustainable chemicals in *Cupriavidus necator*. Metab Eng. 2020;62:207–20. 10.1016/j.ymben.2020.09.002 32961296

[47] Wang X, Wang K, Wang L et al. Engineering *Cupriavidus necator* H16 for heterotrophic and autotrophic production of myo-inositol. Bioresour Technol. 2023;368:128321. 10.1016/j.biortech.2022.128321 36379295

[48] Hanko EKR, Sherlock G, Minton NP et al. Biosensor-informed engineering of *Cupriavidus necator* H16 for autotrophic D-mannitol production. Metab Eng. 2022;72:24–34. 10.1016/j.ymben.2022.02.003 35149227

[49] Pan H, Wang J, Wu H et al. Synthetic biology toolkit for engineering *Cupriavidus necator* H16 as a platform for CO_2_ valorization. Biotechnol Biofuels. 2021;14:212. 10.1186/s13068-021-02063-0 34736496 PMC8570001

[50] Alagesan S, Hanko EKR, Malys N et al. Functional genetic elements for controlling gene expression in *Cupriavidus necator* H16. Appl Environ Microb. 2018;84:e00878–18. 10.1128/AEM.00878-18 PMC6146998 30030234

[51] Nakamura AK, Fulk EM, Johnson CW et al. Synthetic genetic elements enable rapid characterization of inorganic carbon uptake systems in *Cupriavidus necator* H16. ACS Synth Biol. 2025;14:943–53. 10.1021/acssynbio.4c00869 40048245 PMC11934965

[52] Fricke WF, Kusian B, Bowien B. The genome organization of *Ralstonia eutropha* strain H16 and related species of the Burkholderiaceae. J Mol Microbiol Biotechnol. 2009;16:124–35. 10.1159/000142899 18957867

[53] Jahn M, Crang N, Janasch M et al. Protein allocation and utilization in the versatile chemolithoautotroph *Cupriavidus necator*. eLife. 2021;10:e69019. 10.7554/eLife.69019 34723797 PMC8591527

[54] Jahn M, Crang N, Gynnå AH et al. The energy metabolism of *Cupriavidus necator* in different trophic conditions. Appl Environ Microb. 2024;90:e00748–24. 10.1128/aem.00748-24 PMC11540253 39320125

[55] Calvey CH, Sànchez i Nogué V, White AM et al. Improving growth of *Cupriavidus necator* H16 on formate using adaptive laboratory evolution-informed engineering. Metab Eng. 2023;75:78–90. 10.1016/j.ymben.2022.10.016 36368470

[56] Bruch M, Sanchez-Velandia JE, Rodríguez-Pereira J et al. Upcycling atmospheric CO_2_ to polyhydroxyalkanoates via sequential chemo-biocatalytic processes. Green Chem. 2024;26:11885–98. 10.1039/D4GC04228J

[57] Csörgő B, León LM, Chau-Ly IJ et al. A compact Cascade–Cas3 system for targeted genome engineering. Nat Methods. 2020;17:1183–90. 10.1038/s41592-020-00980-w 33077967 PMC7611934

[58] Lynch MD, Gill RT. Broad host range vectors for stable genomic library construction. Biotech Bioeng. 2006;94:151–8. 10.1002/bit.20836 16496398

[59] Chen W, Zhang Y, Zhang Y et al. CRISPR/Cas9-based genome editing in *Pseudomonas aeruginosa* and cytidine deaminase-mediated base editing in Pseudomonas species. iScience. 2018;6:222–31. 10.1016/j.isci.2018.07.024 30240613 PMC6137401

[60] Ling C, Peabody GL, Salvachúa D et al. Muconic acid production from glucose and xylose in *Pseudomonas putida* via evolution and metabolic engineering. Nat Commun. 2022;13:4925. 10.1038/s41467-022-32296-y 35995792 PMC9395534

[61] Wetmore KM, Price MN, Waters RJ et al. Rapid quantification of mutant fitness in diverse bacteria by sequencing randomly bar-coded transposons. mBio. 2015;6:e00306–15. 10.1128/mBio.00306-15 25968644 PMC4436071

[62] Schmidt M, Lee N, Zhan C et al. Maximizing heterologous expression of engineered type I polyketide synthases: investigating codon optimization strategies. ACS Synth Biol. 2023;12:3366–80. 10.1021/acssynbio.3c00367 37851920 PMC10661030

[63] Simon R, Priefer U, Pühler A. A broad host range mobilization system for *in vivo* genetic engineering: transposon mutagenesis in gram negative bacteria. Nat Biotechnol. 1983;1:784–91. 10.1038/nbt1183-784

[64] Blodgett JAV, Thomas PM, Li G et al. Unusual transformations in the biosynthesis of the antibiotic phosphinothricin tripeptide. Nat Chem Biol. 2007;3:480–5. 10.1038/nchembio.2007.9 17632514 PMC4313788

[65] Dolan AE, Hou Z, Xiao Y et al. Introducing a spectrum of long-range genomic deletions in human embryonic stem cells using type I CRISPR-Cas. Mol Cell. 2019;74:936–950. 10.1016/j.molcel.2019.03.014 30975459 PMC6555677

[66] Bi C, Su P, Müller J et al. Development of a broad-host synthetic biology toolbox for *Ralstonia eutropha* and its application to engineering hydrocarbon biofuel production. Microb Cell Fact. 2013;12:107. 10.1186/1475-2859-12-107 24219429 PMC3831590

[67] Xiong B, Li Z, Liu L et al. Genome editing of *Ralstonia eutropha* using an electroporation-based CRISPR-Cas9 technique. Biotechnol Biofuels. 2018;11:172. 10.1186/s13068-018-1170-4 29951116 PMC6011247

[68] Lenz O, Lauterbach L, Frielingsdorf S. O2-tolerant [NiFe]-hydrogenases of *Ralstonia eutropha* H16: physiology, molecular biology, purification, and biochemical analysis. Methods Enzymol. 2018;613:117–51. 10.1016/bs.mie.2018.10.008 30509463

[69] Lv Y, Qian S, Du G et al. Coupling feedback genetic circuits with growth phenotype for dynamic population control and intelligent bioproduction. Metab Eng. 2019;54:109–16. 10.1016/j.ymben.2019.03.009 30940507

[70] Rugbjerg P, Sarup-Lytzen K, Nagy M et al. Synthetic addiction extends the productive life time of engineered *Escherichia coli* populations. Proc Natl Acad Sci USA. 2018;115:2347–52. 10.1073/pnas.1718622115 29463739 PMC5877936

[71] Schulz-Mirbach H, Dronsella B, Erb TJ. *Escherichia coli* selection strains for growth-coupled metabolic engineering. Trends Biotechnol;2026;44:92–110. 10.1016/j.tibtech.2025.06.015 40653433

[72] Martínez-García E, Nikel PI, Chavarría M et al. The metabolic cost of flagellar motion in *Pseudomonas putida* KT2440. Environ Microbiol. 2014;16:291–303. 10.1111/1462-2920.12309 24148021

[73] Pearcy N, Garavaglia M, Millat T et al. A genome-scale metabolic model of *Cupriavidus necator* H16 integrated with TraDIS and transcriptomic data reveals metabolic insights for biotechnological applications. PLoS Comput Biol. 2022;18:e1010106. 10.1371/journal.pcbi.1010106 35604933 PMC9166356

