# Supplementary material for: Cas3-mediated genome reduction: demonstration in Cupriavidus necator H16 improves growth on heterotrophic and autotrophic carbon sources
Source: Nucleic Acids Res. 2026 Aug 4;54(14):gkag753. doi: 10.1093/nar/gkag753 (PMC13434340; doi:10.1093/nar/gkag753)
Supplement: Supplementary file 1 [file gkag753_supplemental_files.zip › Supplemental Information.pdf]

Cas3-mediated genome reduction: Demonstration in *Cupriavidus necator* H16 improves growth on heterotrophic and autotrophic carbon sources

Emily M. Fulk, Reuben M. Swart, Akira K. Nakamura, Laura B. Quinto, Violeta Sànchez i Nogué, Christopher H. Calvey, Indulekha Tharun, Farren J. Isaacs, Christopher W. Johnson

SUPPLEMENTAL INFORMATION

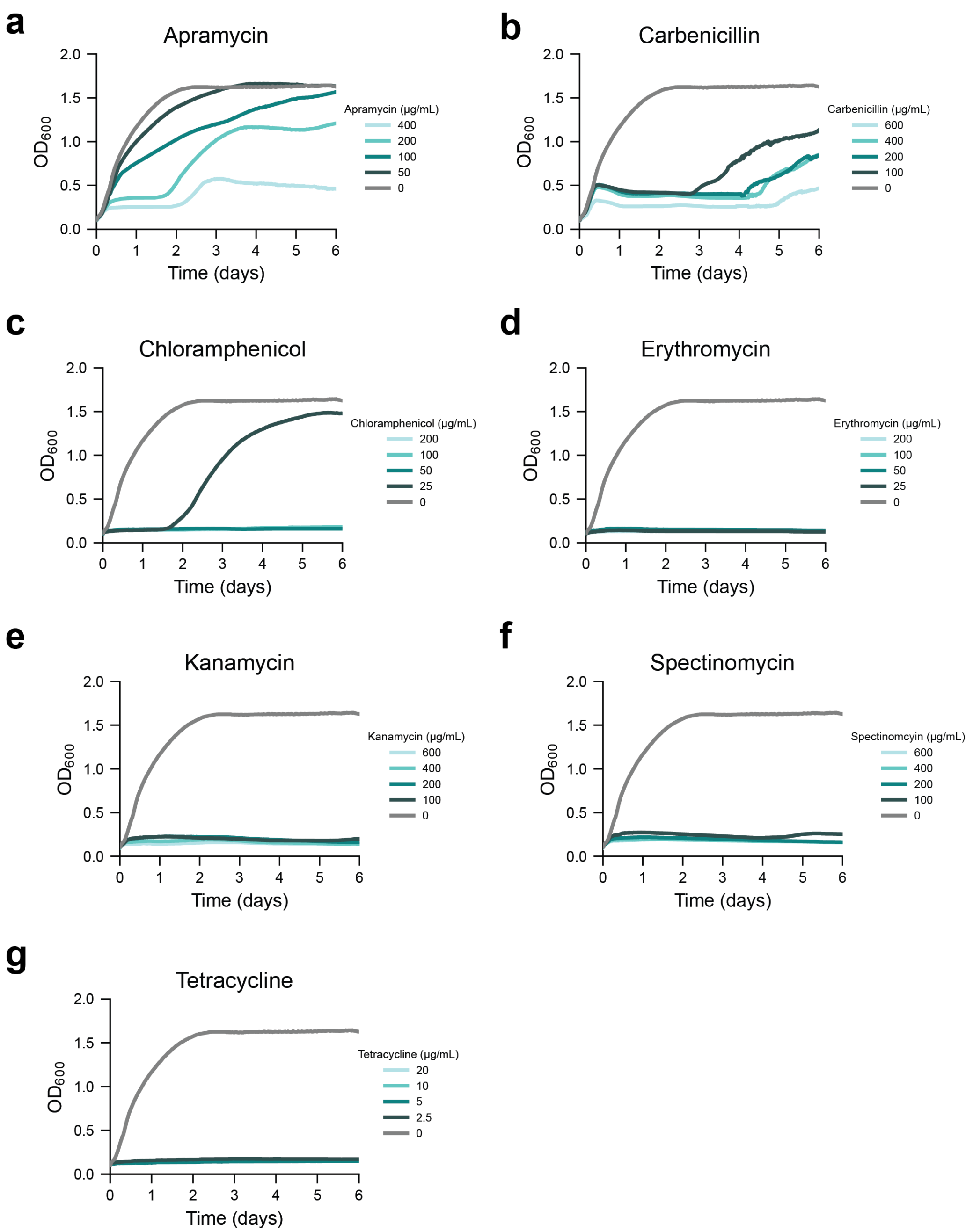

Figure S1. Antibiotic susceptibility of *C. necator*. A parental *C. necator* strain (CHC023) was grown on a 50/50 v/v mix of LB and FN10 medium with varying concentrations of **(a)** apramycin, **(b)** carbenicillin, **(c)** chloramphenicol, **(d)** erythromycin, **(e)** kanamycin, (**f**) spectinomycin, or **(g)** tetracycline in a plate reader. Data represent the average of three biological replicates.

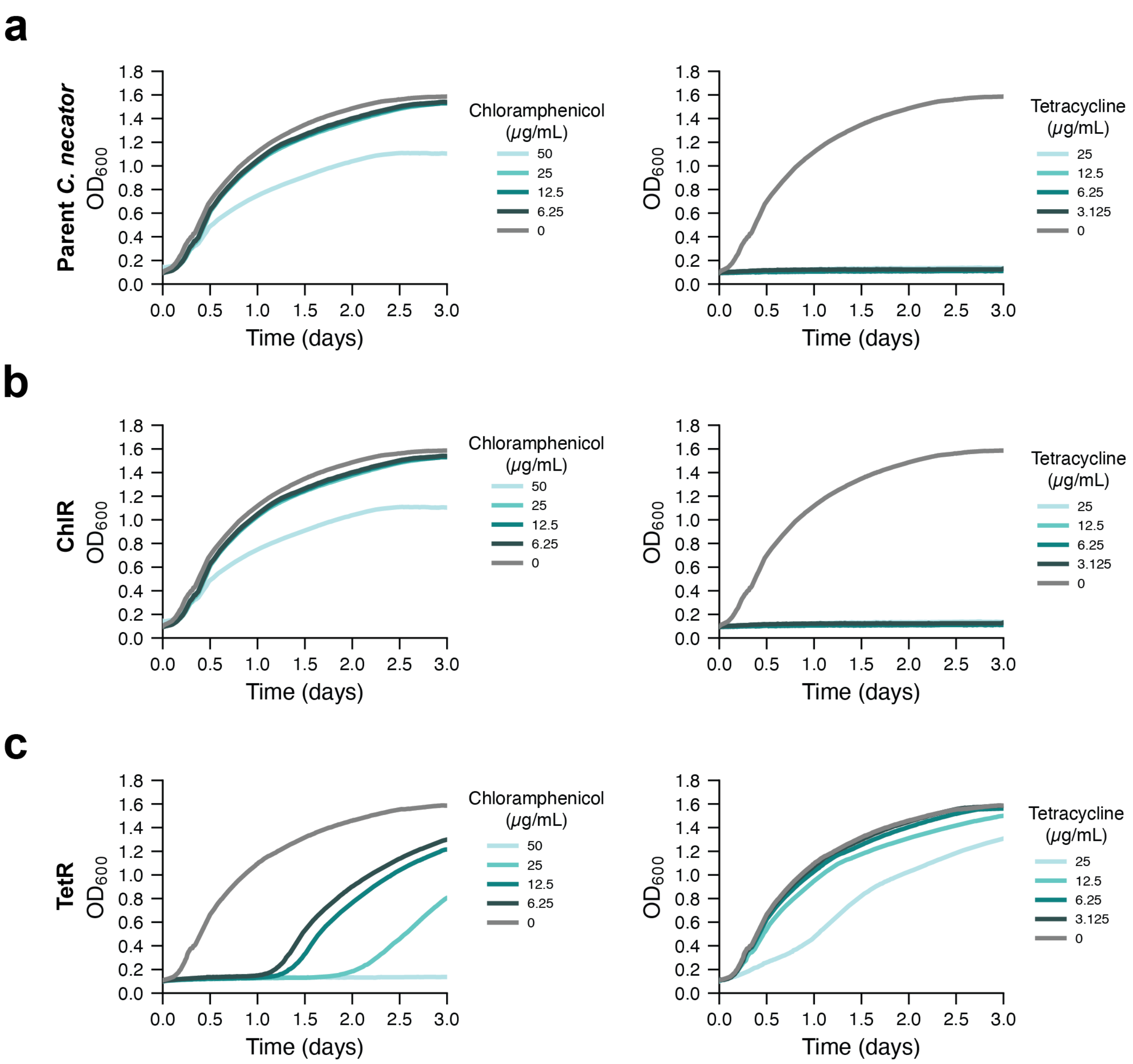

Figure S2. Evaluation of chloramphenicol and tetracycline resistance markers in *C. necator*. (a) Parental *C. necator* (CHC023), **(b)** *C. necator* expressing a chloramphenicol resistance marker (EMF069), and **(c)** *C. necator* expressing a tetracycline resistance marker (EMF070) were grown in a 50/50 v/v mix of LB and FN10 medium with varying concentrations of chloramphenicol or tetracycline in a plate reader. Data represent the average of three biological replicates.

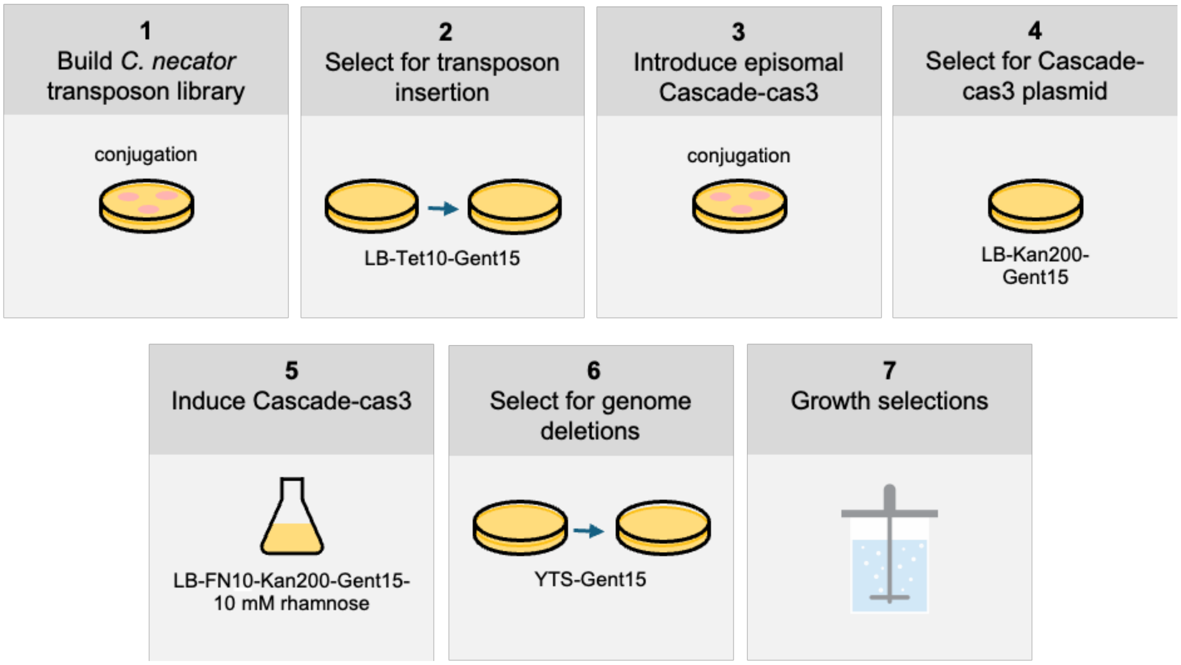

Figure S3. Generating genome-reduced mutant pools. The overall workflow for generating a reduced-genome *C. necator* library using TRIM3 is as follows: (1) A transposon carrying selectable and counter-selectable markers is conjugated into *C. necator*; (2) Selection on tetracycline (to select for *C. necator* with the inserted transposon) and gentamicin (to select against the conjugation donor strain) to generate a transposon library; (3) A replicative plasmid for inducible Cascade-Cas3 and crRNA expression is conjugated into the transposon library; (4) Selection on kanamycin and gentamicin selects for *C. necator* transconjugants carrying the Cascade-Cas3 plasmid; (5) Cascade-Cas3 expression is induced with 10 mM rhamnose; (6) *C. necator* isolates that have lost the counter-selectable marker *sacB* are selected for on yeast-tryptone plates with 15% sucrose; (7) growth selections enrich for mutants containing beneficial mutations in the population. Following selections, long-read nanopore sequencing can identify deletions and other mutations within isolates.

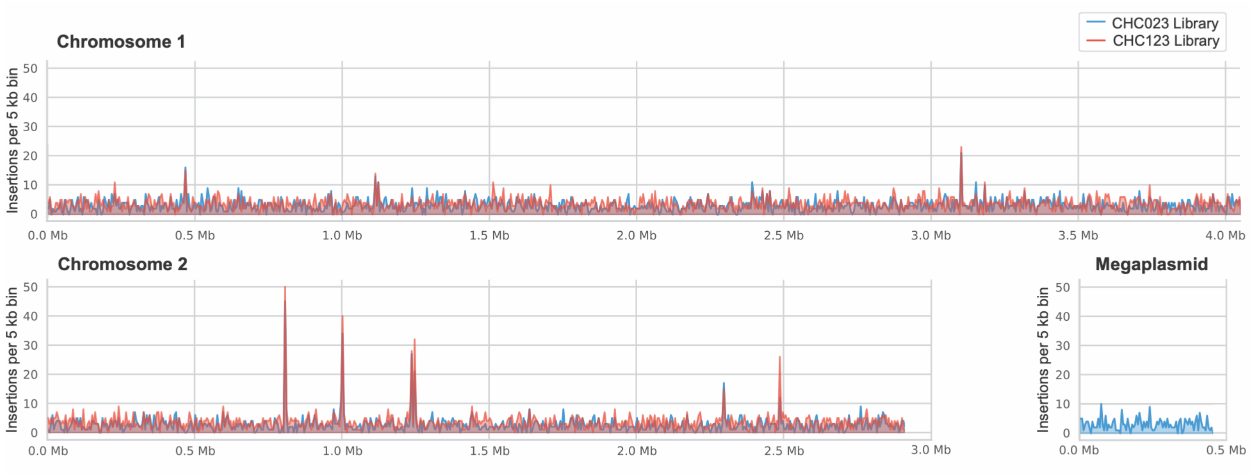

Figure S4. Distribution of transposon insertions across the genome in the CHC023 (blue) and CHC123 (red) libraries. Transposon libraries were subjected to NGS sequencing and the transposon insertions were mapped using scripts and parameters available at https://github.com/akirakaren/map_Tninsertions.

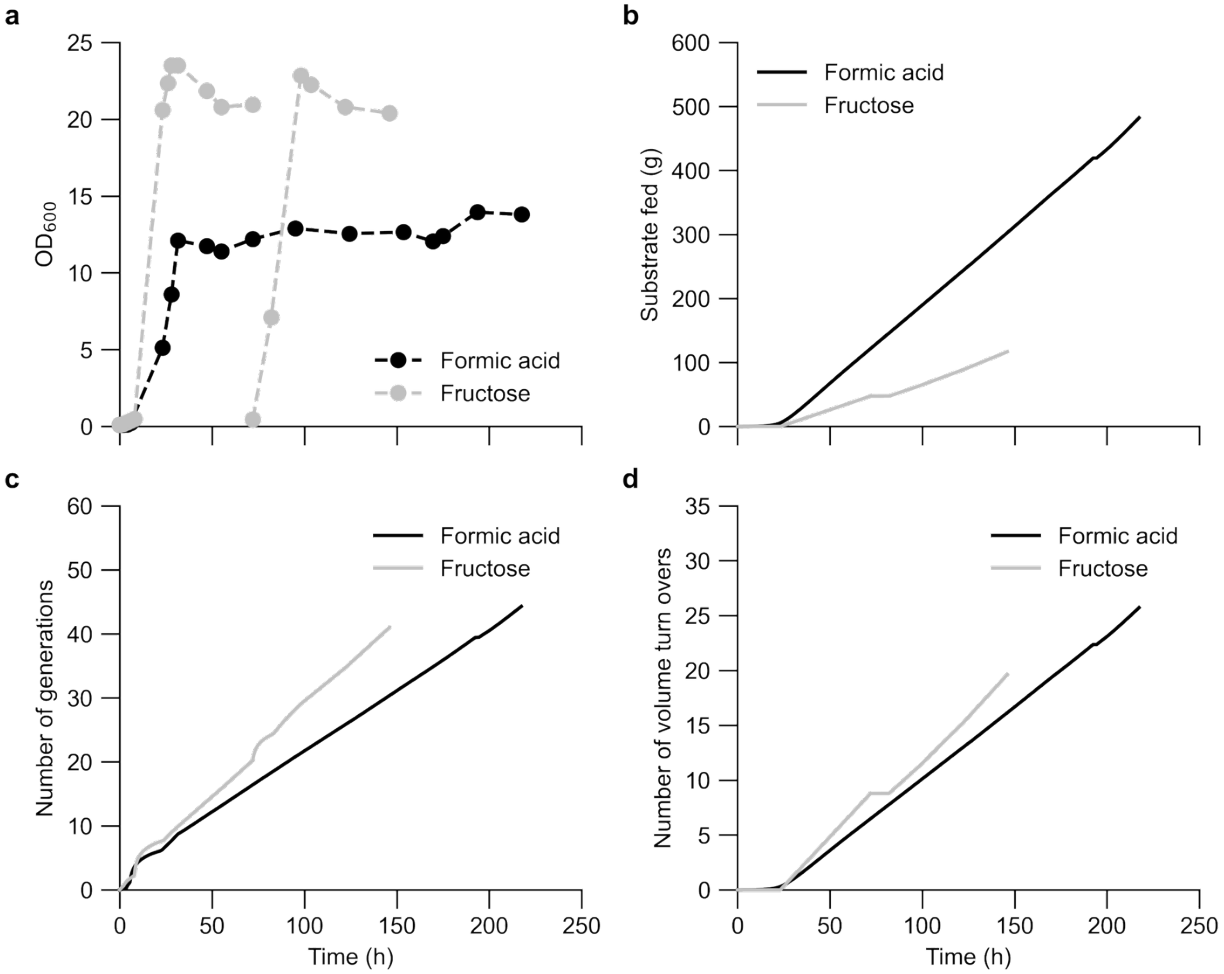

Figure S5. Growth enrichment of reduced-genome CHC023 libraries on formic acid and fructose in bioreactors. (a) OD_{600}, **(b)** mass of substrate fed to bioreactors, **(c)** number of generations, and **(d)** number of bioreactor volume turn overs during growth of reduced-genome CHC023 (*C. necator* H16 ΔRE Δ*phaCAB*) libraries on minimal medium containing 7.5% (w/v) formic acid or 20 g/L fructose as the sole carbon and energy source.

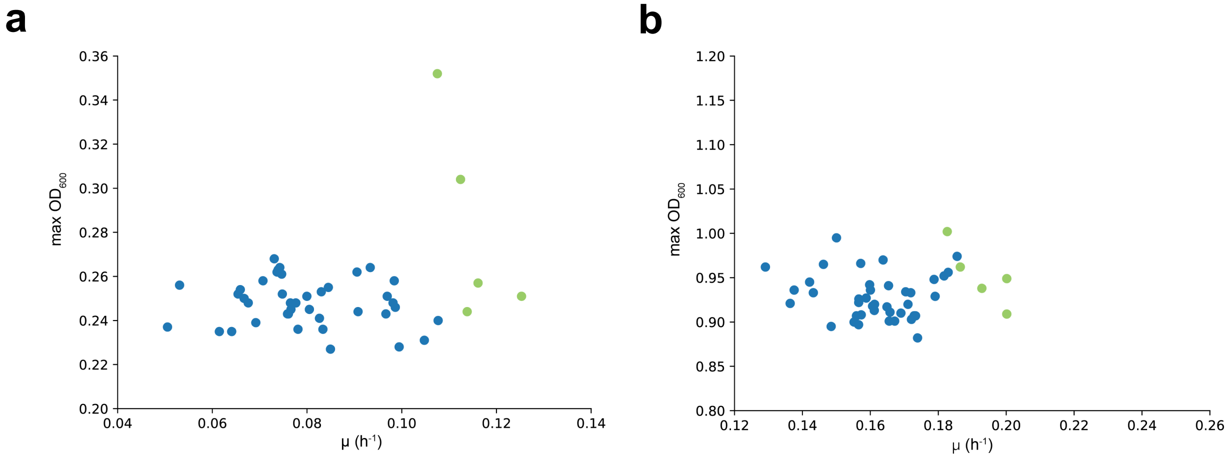

Figure S6. Preliminary evaluation of genome-reduced CHC023 isolates. Growth-selected CHC023 genome-reduced isolates were grown in minimal medium containing **(a)** 4.08 g/L sodium formate or **(b)** 2 g/L fructose as

the sole source of carbon and energy. Estimated growth rates were calculated from the grow curves of individual replicates, and five isolates from each selection (green) were identified for further characterization.

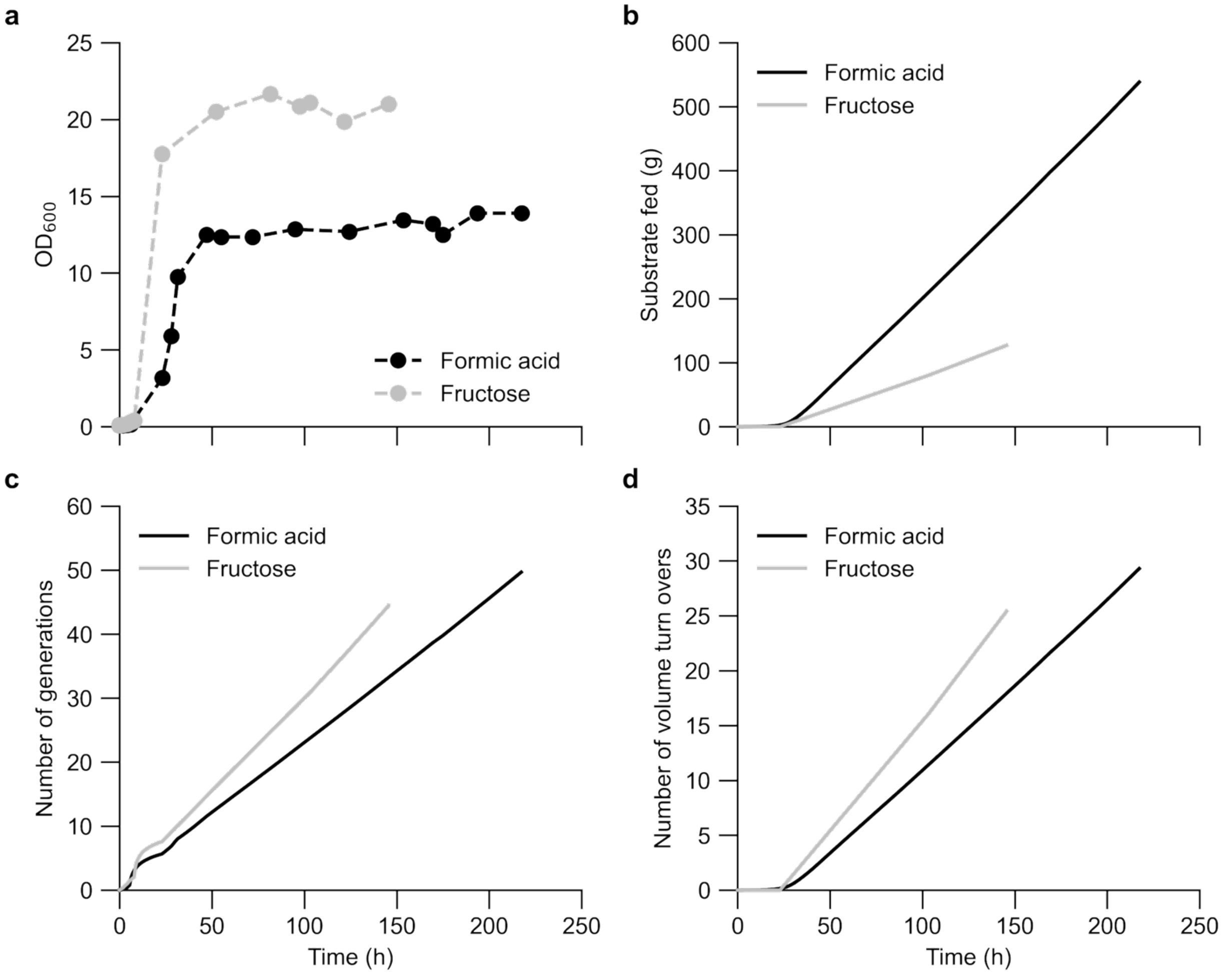

Figure S7. Growth selection of reduced-genome CHC123 libraries on formic acid and fructose in bioreactors. (a) OD_{600}, **(b)** mass of substrate fed to bioreactors, **(c)** number of generations, and **(d)** number of bioreactor volume turn overs during growth of reduced-genome CHC123 (*C. necator* H16 ΔRE Δ*phaCAB* ΔpHG1) libraries on minimal medium containing 7.5 % (w/v) formic acid or 20 g/L fructose as the sole carbon and energy source.

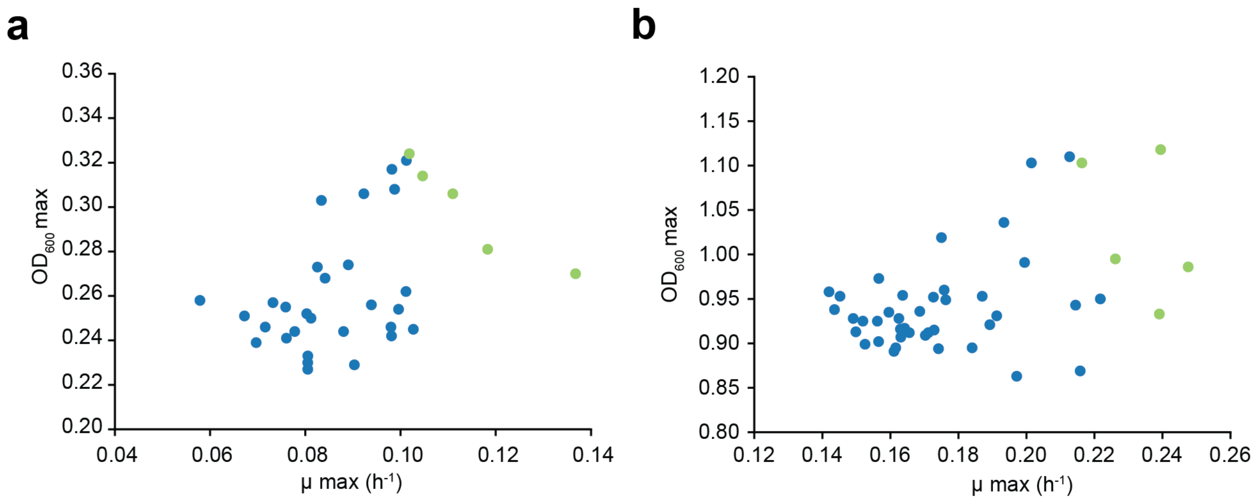

Figure S8. Preliminary evaluation of genome-reduced CHC123 isolates. Growth-selected CHC123 genome-reduced isolates were grown in minimal medium containing **(a)** 4.08 g/L Na-formate or **(b)** 2 g/L fructose as the sole source of carbon and energy. Estimated growth rates were calculated from the grow curves of individual replicates, and five isolates from each selection (green) were identified for further characterization.

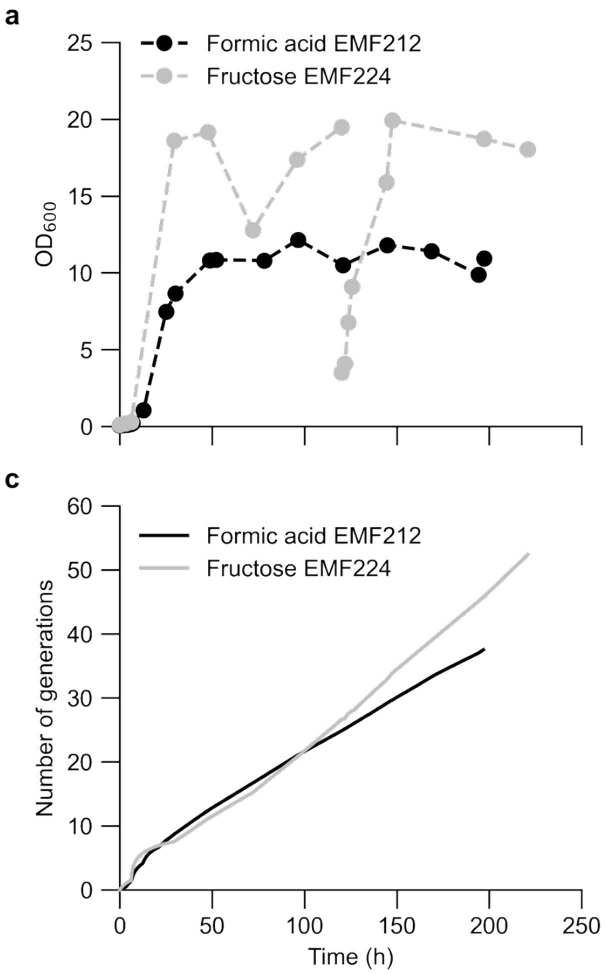

Figure S9. Growth selection of second-generation minimized libraries on formic acid and fructose in bioreactors. (a) OD_{600}, **(b)** mass of substrate fed to bioreactors, **(c)** number of generations, and **(d)** number of bioreactor volume turn overs during growth of second-generation reduced genome libraries of EMF212 grown on 7.5% (w/v) formic acid and EMF224 grown on 20 g/L fructose as the sole carbon and energy source.

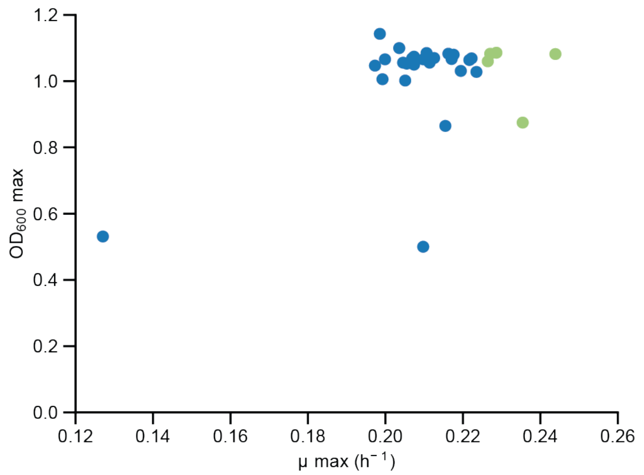

Figure S10. Preliminary evaluation of second generation reduced EMF224 isolates. Growth-selected EMF224 genome-reduced isolates were grown in minimal medium containing 2 g/L fructose as the sole source of carbon and energy. Estimated growth rates were calculated from the growth curves of individual replicates, and five isolates from each selection (green) were identified for further characterization.

Table S1. Deletions observed in growth-selected isolates. Deletions observed in growth-selected isolates that were not in the parent strains CHC023 and CHC123 as mapped to a *C. necator* reference genome (RefSeq GCF_004798725.1). The reduction for each individual deletion is calculated as a percentage of the full-length genome (7.4 Mb), while the total deletion and reduction include deletion of the megaplasmid (pHG1) were applicable. Notes include selected functions within deleted region, where known. "Other mutations" include SNPs and small indels (<200 bp) present in each isolate that were not present in the parent strain (see **Table S2** for more details). "Convergent mutations" constitute other mutations occurring in the same gene in multiple independent isolates. Abbreviations are as follows: Ch1: Chromosome 1; Ch2: chromosome 2; del: deletion; ins: insertion.

Isolate	Deletion ID	Replicon	Deletion start	Deletion end	Deletion size (bp)	Reduction (%)	Notes	Total deletion (bp)	Total reduction (%)	Number of other mutations (Table S2)	Convergent mutations				
											E6A55_ 01580	E6A55_0 3500	*phcR*	*ptsP*	*waaC*
CHC023-derived isolates selected on formic acid (**Figure 2**)															
EMF206	1	pHG1	421826	93025	123337	1.7	CBB operon; hydrogenases	179,413	2.4	3				Q569*	
	2	pHG1	224893	280968	56076	0.8	Denitrification								
EMF207	1	pHG1	421826	93025	123337	1.7	CBB operon; hydrogenases	242,495	3.3	2			12-bp del		10-bp del
	2	pHG1	195082	314241	119158	1.6	Denitrification								
EMF209	1	pHG1	421826	93025	123337	1.7	CBB operon; hydrogenases	231,074	3.1	9		L85P			10-bp del
	2	pHG1	263413	371150	107737	1.5	Denitrification; conjugation								
EMF210	1	pHG1	421826	93025	123337	1.7	CBB operon; hydrogenases	146,098	2.0	2					10-bp del
	2	pHG1	250570	273330	22761	0.3	Denitrification								
CHC123-derived isolates selected on formic acid (**Figure 4**)															
EMF211	1	chr 2	2174959	2286570	111612	1.5	Secondary metabolism	563,751	7.6	2		54-bp del			
EMF212	1	chr 2	2639341	2718714	79374	1.1	Flagella	531,513	7.2	0					
EMF214	1	chr 2	2196223	2289169	92947	1.3	Secondary metabolism	545,086	7.4	1				3-bp ins (repeat)	
EMF215	1	chr 2	2639341	2718714	79374	1.1	Flagella; same deletions as EMF212	531,513	7.2	1		93-bp del			
CHC023-derived isolates selected on fructose (**Figure 3**)															
EMF219	1	pHG1	421826	93025	123337	1.7	CBB operon; hydrogenases	146,098	2.0	2			12-bp del		10-bp del
	2	pHG1	250570	273330	22761	0.3	Denitrification								
EMF220	1	pHG1	421826	93025	123337	1.7	CBB operon; hydrogenases	176,319	2.4	2			12-bp del		10-bp del
	2	pHG1	265771	318753	52982	0.7	Denitrification								
CHC123-derived isolates selected on fructose (**Figure 5**)															
EMF223	1	chr 2	2000974	2601722	600749	8.1	Secondary metabolism	1,052,888	14.2	2			12-bp del		
EMF224	1	chr 2	2045086	2695121	650036	8.8	Secondary metabolism; flagella	1,102,175	14.9	1					
EMF225	1	chr 2	2363417	2656159	292743	3.9	Secondary metabolism	744,882	10.0	2			12-bp del		
EMF224-derived isolates selected on fructose (**Figure 7**)															
EMF248	1	chr 2	2045086	2695121	650036	8.8	Deletion inherited from EMF224	1,103,204	14.9	2	L41Q				
	2	chr 1	161701	162729	1029	0.0	Unknown function								
EMF249	1	chr 2	1902070	2812682	910613	12.3	Deletion from EMF224 extended by 261 kb	1,362,752	18.4	2	R59P				
EMF250	1	chr 2	2045086	2695121	650036	8.8	Deletion inherited from EMF224	1,110,991	15.0	3	E109K				
	2	chr 1	161644	170479	8816	0.1	Methionine biosynthesis; transcriptional regulator								
EMF252	1	chr 2	2045086	2695121	650036	8.8	Deletion inherited from EMF224	1,109,987	15.0	4	L210R				
	2	chr 1	162668	170479	7812	0.1	Methionine biosynthesis; transcriptional regulator								
EMF212-derived isolates selected on formic acid (**Figure 6**)															
EMF258	1	chr 2	2639341	2718714	79374	1.1	Deletion inherited from EMF212	542,598	7.3	1					K98T
	2	chr 2	2598539	2609623	11085	0.1	Secondary metabolism								

Table S2. Other mutations observed in growth-selected isolates. Mutations beyond the large deletions observed in growth-selected isolates that were not in the parent strains CHC023 and CHC123 as mapped to a *C. necator* reference genome (RefSeq GCF_004798725.1). Arrows in Gene column indicate gene direction.

Isolate	Replicon	Position	Mutation	Mutation type	Mutation details	Gene	Origin
CHC023	pHG1	682	C→A	SNP	P190T (CCG→ACG)	E6A55_32200 CDS (twin-arginine translocation signal domain-containing protein) →	ancestor
CHC123	chr1	1862994	(AGAGCTG)17→15	Repeat change	coding (85-98/213 nt)	E6A55_08655 CDS (hypothetical protein) ←	ancestor
EMF206	chr1	348744	C→T	SNP	Q569* (CAG→TAG)	*ptsP* CDS (phosphoenolpyruvate--protein phosphotransferase) →	de novo
	chr1	1638531	T→G	SNP	V80G (GTG→GGG)	E6A55_07620 CDS (transcriptional regulator) →	de novo
	chr2	1587347	Δ17 bp	Small deletion	coding (538-554/897 nt)	E6A55_26340 CDS (tryptophan 2_3-dioxygenase) →	de novo
EMF207	chr1	998222	Δ10 bp	Small deletion		*waaC* CDS (lipopolysaccharide heptosyltransferase I)	de novo
	chr1	3375413	(CGGTGGCGCTCG)2→1	Repeat change	coding (378-389/1146 nt)	*phcR* CDS (hybrid sensor histidine kinase_response regulator) ←	de novo
EMF209	chr1	544657	C→T	SNP	R126H (CGC→CAC)	E6A55_02625 CDS (1-acyl-sn-glycerol-3-phosphate acyltransferase) ←	de novo
	chr1	743733	A→G	SNP	L85P (CTG→CCG)	E6A55_03500 CDS (sensor histidine kinase) ←	de novo
	chr1	998222	Δ10 bp	Small deletion		*waaC* CDS (lipopolysaccharide heptosyltransferase I)	de novo
	chr1	1314606	A→G	SNP	L380P (CTG→CCG)	*mutS* CDS (DNA mismatch repair protein MutS) ←	de novo
	chr1	1778067	(CG)5→4	Repeat change	coding (99-100/1188 nt)	E6A55_08260 CDS (MFS transporter) ←	de novo
	chr2	218161	G→A	SNP	G170D (GGC→GAC)	E6A55_20070 CDS (sigma-54-dependent Fis family transcriptional regulator) →	de novo
	chr2	798505	(GC)5→4	Repeat change	coding (747-748/1197 nt)	E6A55_22730 CDS (helix-turn-helix transcriptional regulator) ←	de novo
	chr2	1537195	A→G	SNP	E339G (GAG→GGG)	E6A55_26120 CDS (long-chain fatty acid--CoA ligase) →	de novo
	chr2	1804313	T→C	SNP	Y173H (TAC→CAC)	E6A55_27270 CDS (extracellular solute-binding protein) →	de novo
EMF210	chr1	402067	C→T	SNP	A274T (GCC→ACC)	E6A55_01940 CDS (HPr kinase_phosphorylase) ←	de novo
	chr1	998222	Δ10 bp	Small deletion		*waaC* CDS (lipopolysaccharide heptosyltransferase I)	de novo
EMF211	chr1	742813	Δ54 bp	Small deletion	coding (1121-1174/1950 nt)	E6A55_03500 CDS (sensor histidine kinase) ←	de novo
	chr2	1984021	A→C	SNP	D457A (GAC→GCC)	E6A55_28005 CDS (cyclohexanone monooxygenase) →	de novo
EMF214	chr1	348402	(TGC)4→5	Repeat change	coding (1363/1761 nt)	*ptsP* CDS (phosphoenolpyruvate--protein phosphotransferase) →	de novo
EMF215	chr1	742828	Δ93 bp	Small deletion	coding (1067-1159/1950 nt)	E6A55_03500 CDS (sensor histidine kinase) ←	de novo
EMF219	chr1	998222	Δ10 bp	Small deletion		*waaC* CDS (lipopolysaccharide heptosyltransferase I)	de novo
	chr1	3375413	(CGGTGGCGCTCG)2→1	Repeat change	coding (378-389/1146 nt)	*phcR* CDS (hybrid sensor histidine kinase_response regulator) ←	de novo
EMF220	chr1	998222	Δ10 bp	Small deletion		*waaC* CDS (lipopolysaccharide heptosyltransferase I)	de novo
	chr1	3375413	(CGGTGGCGCTCG)2→1	Repeat change	coding (378-389/1146 nt)	*phcR* CDS (hybrid sensor histidine kinase_response regulator) ←	de novo
EMF223	chr1	1862994	(AGAGCTG)17→15	Repeat change	coding (85-98/213 nt)	E6A55_08655 CDS (hypothetical protein) ←	CHC123 parent
	chr1	3375413	(CGGTGGCGCTCG)2→1	Repeat change	coding (378-389/1146 nt)	*phcR* CDS (hybrid sensor histidine kinase_response regulator) ←	de novo
EMF224	chr1	3401734	G→T	SNP	T6K (ACG→AAG)	E6A55_16020 CDS (LysR family transcriptional regulator) ←	de novo
EMF225	chr1	1862994	(AGAGCTG)17→15	Repeat change	coding (85-98/213 nt)	E6A55_08655 CDS (hypothetical protein) ←	CHC123 parent
	chr1	3375413	(CGGTGGCGCTCG)2→1	Repeat change	coding (378-389/1146 nt)	*phcR* CDS (hybrid sensor histidine kinase_response regulator) ←	de novo
EMF248	chr1	328569	A→T	SNP	L41Q (CTG→CAG)	E6A55_01580 CDS (GntR family transcriptional regulator) ←	de novo
	chr1	3401734	G→T	SNP	T6K (ACG→AAG)	E6A55_16020 CDS (LysR family transcriptional regulator) ←	EMF224 parent
EMF249	chr1	328515	C→G	SNP	R59P (CGG→CCG)	E6A55_01580 CDS (GntR family transcriptional regulator) ←	de novo
	chr1	3401734	G→T	SNP	T6K (ACG→AAG)	E6A55_16020 CDS (LysR family transcriptional regulator) ←	EMF224 parent
EMF250	chr1	328366	C→T	SNP	E109K (GAA→AAA)	E6A55_01580 CDS (GntR family transcriptional regulator) ←	de novo
	chr1	1862994	(AGAGCTG)17→15	Repeat change	coding (85-98/213 nt)	E6A55_08655 CDS (hypothetical protein) ←	CHC123 grandparent
	chr1	3401734	G→T	SNP	T6K (ACG→AAG)	E6A55_16020 CDS (LysR family transcriptional regulator) ←	EMF224 parent
EMF252	chr1	328062	A→C	SNP	L210R (CTG→CGG)	E6A55_01580 CDS (GntR family transcriptional regulator) ←	de novo
	chr1	400106	Δ1 bp	Small deletion	coding (151/660 nt)	E6A55_01925 CDS (ATP-dependent protease) ←	de novo
	chr1	1862994	(AGAGCTG)17→15	Repeat change	coding (85-98/213 nt)	E6A55_08655 CDS (hypothetical protein) ←	CHC123 grandparent
	chr1	3401734	G→T	SNP	T6K (ACG→AAG)	E6A55_16020 CDS (LysR family transcriptional regulator) ←	EMF224 parent
EMF258	chr1	997931	T→G	SNP	K98T (AAG→ACG)	*waaC* CDS (lipopolysaccharide heptosyltransferase I) ←	de novo

Table S3. Deletion junctions in growth-selected isolates. Sequence remaining after repair of excision is in upper case text. Deleted sequence is in lower case text. Potential microhomology near the repaired junctions is in bold text. Boundaries may not be precise due to variability in possible alignment.

Isolate	Deletion ID	Replicon	Deletion start	Deletion end	Upstream junction	Downstream junction
EMF206	1	pHG1	421826	93025	...TGAGCCAGGTATGCAG**TTTTg**tcgcttatgtctctctctc...	...acggcattgacgacaattccGGCTC**TTTTG**AAAAATGAGT...
	2	pHG1	224893	280968	...TGTGAGCAGAAGATCAT**GGC**ccagtcggcgccgcagccca...	...gacgtcgatcggcttgccagC**GGC**ACTGTCCGGTGCCGCG...
EMF207	1	pHG1	421826	93025	...TGAGCCAGGTATGCAG**TTTTg**tcgcttatgtctctctctc...	...acggcattgacgacaattccGGCTC**TTTTG**AAAAATGAGT...
	2	pHG1	195082	314241	...ATTGCGTCGGAAC**CACTG**GAtagagcggctcgaggcgagg...	...aaaggaggggggtggccgt**caCTG**ACTCTGCTACCGAAGCG...
EMF209	1	pHG1	421826	93025	...TGAGCCAGGTATGCAG**TTTTg**tcgcttatgtctctctctc...	...acggcattgacgacaattccGGCTC**TTTTG**AAAAATGAGT...
	2	pHG1	263413	371150	...TGCTGGGTTCGTCCAGGAACagcacgcgcggcgaacccag...	...agaacgcgacgacggccccgCTCGATCTTGATCTCATCGT...
EMF210	1	pHG1	421826	93025	...TGAGCCAGGTATGCAG**TTTTg**tcgcttatgtctctctctc...	...acggcattgacgacaattccGGCTC**TTTTG**AAAAATGAGT...
	2	pHG1	250570	273330	...GAGATCGCGCAGGCCTA**CGG**catctccagggcgcatctgg...	...gtgcagggtgccgtcctgct**CGG**TCCAGCGCACCGCGTGC...
EMF211	1	chr 2	2174959	2286570	...CGATAAACCAGGGGATA**CAG**atcgaaacccttgtagctgg...	...aggaatattcctgcccgtc**cAG**CCCCAGAGCATGTGAAAA...
EMF212	1	chr 2	2639341	2718714	...TACAGCAGTGGG**CCCAG**GGCgatgccgaaggcgggcatcg...	...gctgcgggcggatca**cccag**TCCTGGATCTCGCGCAGGTC...
EMF214	1	chr 2	2196223	2289169	...GCCCGGCACTTCCCAAAGA**Tt**gaccgtgcccacatgcagc...	...gccgaccgtgatcgccatg**tT**CAAGAATGTCGCTCAGTGC...
EMF215	1	chr 2	2639341	2718714	...TACAGCAGTGGG**CCCAG**GGCgatgccgaaggcgggcatcg...	...gctgcgggcggatca**cccag**TCCTGGATCTCGCGCAGGTC...
EMF219	1	pHG1	421826	93025	...TGAGCCAGGTATGCAG**TTTTg**tcgcttatgtctctctctc...	...acggcattgacgacaattccGGCTC**TTTTG**AAAAATGAGT...
	2	pHG1	250570	273330	...GAGATCGCGCAGGCCTA**CGG**catctccagggcgcatctgg...	...gtgcagggtgccgtcctgct**CGG**TCCAGCGCACCGCGTGC...
EMF220	1	pHG1	421826	93025	...TGAGCCAGGTATGCAG**TTTTg**tcgcttatgtctctctctc...	...acggcattgacgacaattccGGCTC**TTTTG**AAAAATGAGT...
	2	pHG1	265771	318753	...GCACCATGCAATCCAGG**CAGt**gcaggcattcgcgatggtc...	...caaacaggccgagagtcattCT**CAGT**CTCCTTTAGAAAGC...
EMF223	1	chr 2	2000974	2601722	...TATTGCGGCGGATATACA**GG**ataaagaccgccggcagcac...	...cgccgatggaggcggcgatgC**GG**TCCGTGGTTTCGTCCTT...
EMF224	1	chr 2	2045086	2695121	...CGGGCCATCATGTTCAGTGC**ac**cctacagcgcgttcgagc...	...catctcactaagggtg**ac**gaAAAAAAACTGCGCGACATTC...
EMF225	1	chr 2	2363417	2656159	...GAAGGTGGCTGTTCTTGTTGt**tg**ccatgcgattcggctgt...	...ggtgggacgccggtcggcaa**TG**GCCTGGGCCGTGAGCGCC...
EMF248	1	2045086	2695121	650036	...CGGGCCATCATGTTCAGTGC**ac**cctacagcgcgttcgagc...	...catctcactaagggtg**ac**gaAAAAAAACTGCGCGACATTC...
	2	161701	162729	1029	...TCCCGCCGGTCACCCCAAG**Ttt**ggaggtaaagccgcggcg...	...gatacgccgcaggcccgcc**tTT**CCAGAGTCCCCGCCATGA...
EMF249	1	1902070	2812682	910613	...GTGCGGCTGAGATCGAGCG**Cgc**tgccggccgcctcggctt...	...tgcagcgacaggatcaacgc**CGC**ATAGAGCAGCGTGGCAA...
EMF250	1	2045086	2695121	650036	...CGGGCCATCATGTTCAGTGC**ac**cctacagcgcgttcgagc...	...catctcactaagggtg**ac**gaAAAAAAACTGCGCGACATTC...
	2	161644	170479	8816	...AATTCATGTAAGGCAGAAA**Atc**gtggcaacgaggtgaccc...	...catcgccctgatcgccgcg**aTC**TTCGGCTTCGGCGGAATT...
EMF252	1	2045086	2695121	650036	...CGGGCCATCATGTTCAGTGC**ac**cctacagcgcgttcgagc...	...catctcactaagggtg**ac**gaAAAAAAACTGCGCGACATTC...
	2	chr 2	2639341	2718714	...AAAATCGTCCC**TCTT**TGGCCagcataggccacagcagcgg...	...catcgccctgatcgccgcga**TCTT**CGGCTTCGGCGGAATT...
EMF258	1	chr 2	2598539	2609623	...TACAGCAGTGGG**CCCAG**GGCgatgccgaaggcgggcatcg...	...gctgcgggcggatca**cccag**TCCTGGATCTCGCGCAGGTC...
	2	chr 2	2639341	2718714	...GCTGCTGGCGGGCCTG**GCGCc**cggcattggcgccttgctg...	...gcgtctgccgctgacgcacc**GCGCC**GAAGCGCCCGGCACC...

Supplementary methods

Evaluating antibiotic suitability

To evaluate the sensitivity of parental *C. necator* to various antibiotics, triplicate CHC023 cultures were cultivated overnight in LB+FN10 medium supplemented with 15 µg/mL gentamicin at 30°C, 225 rpm shaking. After ≈16 hours incubation, overnight cultures were spun down, and the cell pellets washed once in medium without antibiotics. The resuspended cell pellets were used to inoculate 200 µL of LB+FN10 medium containing varying concentrations of antibiotics in a 100-well plate for incubation in a BioscreenC microplate reader as previously described (see *Methods*). To evaluate the function of chloramphenicol (*cat*) and tetracycline (*tetA*) resistance markers, *C.* necator

strains containing genomically-integrated *cat* (EMF069) or *tetA* (EMF070) markers were evaluated for growth on varying concentrations of chloramphenicol or tetracycline using the same protocol as described above.

Preliminary evaluation of genome-reduced isolates

To select genome-reduced isolates for further characterization, populations from the end of each growth selection were plated on LB-Gent15 plates and incubated for 48-72 hours. From these plates, individual colonies were picked and inoculated into media containing the same carbon source as the growth selection, either MSM containing 2 g/L fructose and Gent15 or MSM containing 4.08 g/L sodium formate, 100 mM phosphate, and Gent15. 200 μL of each individual replicate was incubated in a microplate in the Bioscreen C at the same settings as previous described for ≈24 hours before being diluted 1:100 into 200 μL fresh medium. These cultures were incubated under the same conditions and maximum growth rates for individual isolates were calculated as previously described (see *Methods*).
